# Intramuscular injection of mesenchymal stem cells activates anabolic and catabolic systems in mouse skeletal muscle

**DOI:** 10.1038/s41598-021-00627-6

**Published:** 2021-10-27

**Authors:** Junya Takegaki, Kohei Sase, Yusuke Kono, Daiki Nakano, Takuya Fujita, Satoshi Konishi, Satoshi Fujita

**Affiliations:** 1 Research Organization of Science and Technology, Ritsumeikan University, 1-1-1 Noji-Higashi, Kusatsu, Shiga 525-8577 Japan; 2 Faculty of Sport and Health Science, Ritsumeikan University, 1-1-1 Noji-Higashi, Kusatsu, Shiga 525-8577 Japan; 3 Ritsumeikan Global Innovation Research Organization, Ritsumeikan University, 1-1-1 Noji-Higashi, Kusatsu, Shiga 525-8577 Japan; 4 College of Pharmaceutical Sciences, Ritsumeikan University, 1-1-1 Noji-Higashi, Kusatsu, Shiga 525-8577 Japan; 5 Faculty of Science and Engineering, Ritsumeikan University, 1-1-1 Noji-Higashi, Kusatsu, Shiga 525-8577 Japan

**Keywords:** Metabolism, Endocrinology

## Abstract

Skeletal muscle mass is critical for good quality of life. Mesenchymal stem cells (MSCs) are multipotent stem cells distributed across various tissues. They are characterized by the capacity to secrete growth factors and differentiate into skeletal muscle cells. These capabilities suggest that MSCs might be beneficial for muscle growth. Nevertheless, little is known regarding the effects on muscle protein anabolic and catabolic systems of intramuscular injection of MSCs into skeletal muscle. Therefore, in the present study, we measured changes in mechanistic target of rapamycin complex 1 (mTORC1) signaling, the ubiquitin–proteasome system, and autophagy-lysosome system-related factors after a single intramuscular injection of MSCs with green fluorescence protein (GFP) into mouse muscles. The intramuscularly-injected MSCs were retained in the gastrocnemius muscle for 7 days after the injection, indicated by detection of GFP and expression of platelet-derived growth factor receptor-alpha. The injection of MSCs increased the expression of satellite cell-related genes, activated mTORC1 signaling and muscle protein synthesis, and increased protein ubiquitination and autophagosome formation (indicated by the expression of microtubule-associated protein 1 light chain 3-II). These results suggest that the intramuscular injection of MSCs activated muscle anabolic and catabolic systems and accelerated muscle protein turnover.

## Introduction

Skeletal muscle is critical for maintaining activities of daily living. The age-related loss of muscle mass and function, i.e., sarcopenia, increases the risk of falls and is associated with type-2 diabetes and cardiovascular diseases^1–3^. Adequate nutritional intake and exercise attenuate or ameliorate sarcopenia^4^. By contrast, aging induces anabolic resistance to protein intake and resistance exercise, making it difficult for older individuals to maintain or increase muscle mass^5^. Therefore, an alternative modality to induce muscle anabolism is necessary to prevent sarcopenia.

Skeletal muscle mass is controlled by the balance between protein synthesis and breakdown. An anabolic stimulus such as resistance exercise or amino acid intake transiently activates muscle protein synthesis, resulting in the accumulation of contractile proteins and muscle hypertrophy^6–8^. The mechanistic target of rapamycin (mTOR) is the crucial regulator of protein synthesis and muscle hypertrophy; numerous anabolic stimuli including resistance exercise, amino acids, and growth factors activate mTOR complex 1 (mTORC1)^9–12^. Although the regulation of protein synthesis and muscle hypertrophy by mTOR is not fully understood, the activation of mTORC1 is essential for muscle hypertrophy both in electrical stimulation and mechanical overload models^13,14^. The ubiquitin–proteasome system and the autophagy-lysosome system primarily mediate muscle protein degradation. The former degrades contractile proteins including myosin heavy chain, myosin light chain, and actin^15–17^, and the latter carries out bulk degradation of cellular components^18^. The mTORC1 and upstream Akt also downregulates two catabolic systems^19,20^. These findings suggest that activating mTORC1 would be critical for maintaining and improving skeletal muscle mass.

Mesenchymal stem cells (MSCs) are multipotent stem cells that are increasingly used in regenerative therapies. MSCs promote the regeneration of skeletal muscle following various types of injury^21–25^. This effect is supported by their capacity to secrete growth factors and differentiate into skeletal muscle cells^26–29^. A study reported that the combination of intramuscular injection of MSCs and downhill-running exercise training evoked skeletal muscle hypertrophy in mice^30^. Another study reported that the intravenous injection of MSCs attenuated high fat diet-induced skeletal muscle atrophy in mice^31^. These results suggest that MSCs have both anabolic and anti-catabolic potencies in skeletal muscle. Nevertheless, the independent effect of MSCs on muscle anabolic and catabolic systems is not fully understood, and elucidating these responses would expand the applicability of MSCs to exercise or nutritional interventions.

To obtain insights into the applicability of the MSCs within skeletal muscle, we measured the transient effects of a single intramuscular injection of MSCs on the muscle anabolic and catabolic systems in the mouse. Because MSCs secrete insulin-like growth factor-1 (IGF-1) that strongly stimulates mTORC1^12,32^, we hypothesized that the intramuscular injection of MSCs would activate mTORC1 signaling and attenuate the ubiquitin–proteasome and autophagy-lysosome systems.

## Materials and methods

### Animals

Twenty-four male C57BL/6J mice (10 weeks old) were obtained from Shimizu Laboratory Supplies (Kyoto, Japan). All animals were housed at 23 ± 1 °C under a 12-h/12-h light–dark cycle and provided food and water ad libitum. All experiments were approved by the Ethics Committee for Animal Experiments at Ritsumeikan University (BKC2018-046) and performed according to Guide for the Care and Use of Laboratory Animals published by the United States National Institutes of Health and the ARRIVE guidelines.

### Cell culture and intramuscular injection

C57BL/6 mouse bone marrow-derived MSCs with GFP were purchased from KAC Co., Ltd. (Kyoto, Japan). MSCs were cultured in Mouse Mesenchymal Stem Cell Culture Medium (Cyagen Biosciences Inc.) at 37 °C in 5% CO_2_. We injected 2.0 × 10^6^ MSCs into the right gastrocnemius muscle of each mouse. The left gastrocnemius muscle was kept intact and served as the control. At 2, 7, 14, and 28 days after MSCs injection, the mice were anesthetized and euthanatized by cervical dislocation, and both gastrocnemius muscles were collected. Three hours before sacrifice, the mice were fasted. Collected muscles were frozen at − 80 °C until use. We previously confirmed that the injection of the vehicle only (phosphate-buffered saline, PBS) did not show similar changes in the typical protein anabolic and catabolic systems to the present study (view Fig. S1).

### Muscle protein synthesis

Muscle protein synthesis was measured using the SUnSET method^33^. Briefly, 0.04 μmol/g bodyweight puromycin diluted in PBS was intraperitoneally injected into each mouse under anesthesia. Fifteen minutes after the injection, the gastrocnemius muscles were removed. Following homogenization, samples were centrifuged at 2000×*g* for 3 min at 4 °C, and the supernatants were collected and processed for western blotting using an anti-puromycin antibody (MABE343, Merck Millipore, Burlington, MA, USA) as described below. The densitometric analysis was performed between 225 and 15 kDa.

### Western blotting

Muscle samples were homogenized and analyzed as previously described with slight modifications^34^. Briefly, samples were homogenized in RIPA buffer (Cell Signaling Technology (CST), Danvers, MA, USA) containing cOmplete Mini protease inhibitor cocktail and PhosSTOP phosphatase inhibitor cocktail (Sigma-Aldrich, St. Louis, MO, USA) and centrifuged at 10,000×*g* for 10 min at 4 °C. The supernatants were collected, and the protein concentration of each sample was determined using a Protein Assay Rapid Kit Wako II (FUJIFILM Wako Pure Chemical, Osaka, Japan). Samples were diluted in 3 × Blue Loading Buffer (CST, Danvers, MA, USA) and boiled at 95 °C for 5 min. Equal amounts of proteins were then separated on 7.5, 10, or 12% TGX gels (BioRad, Hercules, CA, USA) and subsequently transferred to polyvinylidene difluoride membranes. After separating the membranes to ensure that each solution spread throughout the membrane, they were blocked with Bullet Blocking One for Western Blotting (Nacalai Tesque, Kyoto, Japan) for 5 min at room temperature and subsequently incubated overnight at 4 °C with the following primary antibodies: GFP (#2956, CST), platelet-derived growth factor receptor alpha (PDGFRα, #3174, CST), phosphor-Akt (Ser473, #9275, CST), total Akt (#4691, CST), phosphor-p70S6K (Thr389, #9234, CST), total-p70S6K (#2708, CST), phosphor-rpS6 (Ser240/244, #2215, CST), total-rpS6 (#2217, CST), phosphor-4EBP1 (Thr37/46, #9459, CST), total-4EBP1 (#9644, CST), ubiquitinated proteins (#3936, CST), K48-linkage-specific polyubiquitin (#8081, CST), LC3 (#2775, CST), p62/SQSTM (PM045, Medical & Biological Laboratory, Nagoya, Japan), phospho-ERK1/2 (Thr202/Tyr204, #4376, CST), and total-ERK1/2 (#4696, CST). After washing, membranes were incubated for 1 h with the appropriate secondary antibodies at room temperature and visualized using Immobilon Forte Western HRP Substrate (Millipore, CA, USA). Bands were detected using FUSION Chemiluminescence Imaging System (M&S Instruments, Osaka, Japan). Ponceau S staining was used to verify equal loading between lanes and normalization. Band intensities were quantified with Image J software (National Institutes of Health, Bethesda, MD, USA).

### Histological analysis

We created 7-μm sections, and GFP was observed without staining. Some sections were used for hematoxylin and eosin staining, and the others were used for immunohistochemistry.

Immunohistochemical observations were performed as described in a previous study with slight modification^35^. Cryosections were fixed with acetone for 15 min at − 20 °C and blocked with 5% goat serum for 60 min at room temperature. Following blocking, anti-laminin (SAB4200719, Sigma-Aldrich, St. Louis, MO) and anti-LC3 primary antibodies (#2775, CST) in PBS containing 0.1% bovine serum albumin were applied overnight at 4 °C. After washing, sections were incubated for 2 h with goat anti-mouse and anti-rabbit fluorescent-conjugated secondary antibodies at room temperature. Finally, the sections were soaked in VECTASHIELD Vibrance Antifade Mounting Medium (H-1700, Vector Laboratories, Burlingame, CA) and observed using a BZ-9000 microscope (Keyence Co., Osaka, Japan).

### RNA extraction and real-time qPCR

According to the manufacturer's instructions, total RNA was extracted from muscle samples using TRIzol reagent (Invitrogen, Carlsbad, CA, USA). RNA concentrations were measured using a NanoDrop 2000 (Thermo Fisher Scientific, Waltham, MA, USA); 1.5 μg of total RNA was reverse-transcribed into cDNA using a High Capacity cDNA RT kit (Applied Biosystems, Foster City, CA, USA). Gene expression levels were quantified using THUNDERBIRD SYBR qPCR Mix (Toyobo, Osaka, Japan) with a 7500 Fast Sequence Detection System (Applied Biosystems). The gene expressions were quantified using the calibration curve method, and *18 s* was used as a control housekeeping gene. Table 1 shows the sequences of the primers used in this study.

**Table 1:** Primer sequences for qPCR.

Gene	Forward primer (5′–3′)	Reverse primer (5′–3′)
*Pax7*	GTGCCCTCAGTGAGTTCGATTAGC	CCACATCTGAGCCCTCATCCA
*Myod*	TGGCATGATGGATTACAGCG	GAGATGCGCTCCACTATGCT
*Myogenin*	TCCCAACCCAGGAGATCATT	TCAGTTGGGCATGGTTTCGT
*Murf-1*	AGTGTCCATGTCTGGAGGTCGTTT	ACTGGAGCACTCCTGCTTGTAGAT
*Atrogin-1*	TGAGCGACCTCAGCAGTTAC	TTCTCTTCTTGGCTGCGACG
*Musa1*	TCGTGGAATGGTAATCTTGC	CCTCCCGTTTCTCTATCACG
*Trim32*	GTGGACTCGCGTCGGAGCTG	GGTTCAGGTGAGAAGCTGCTGC
*Nedd4*	GTGGGAAGAGAGGCAGGATGTC	GCGAATTCACAGGAAGTGTAGGC
*Ozz*	CTATCACACGCCACCACAAC	GCAGAAGAGAACACCCAAGC
*Igf-1*	CAAGCCCACAGGCTATGGC	TCTGAGTCTTGGGCATGTCAG
*18 s*	CCTGGATACCGCAGCTAGGA	GCGGCGCAATACGAATGCCCC

### Statistical analysis

For comparisons of the GFP protein expression, a *t*-test was used. For comparisons of the other items, two-way ANOVA (MSCs × time) was used. If an interaction was observed, Bonferroni multiple-comparison testing was performed. All values were expressed as mean ± SEM with individual plots. Statistical significance of differences was defined as *P* < 0.05.

## Results

### Markers of MSCs

**Figure 1:**
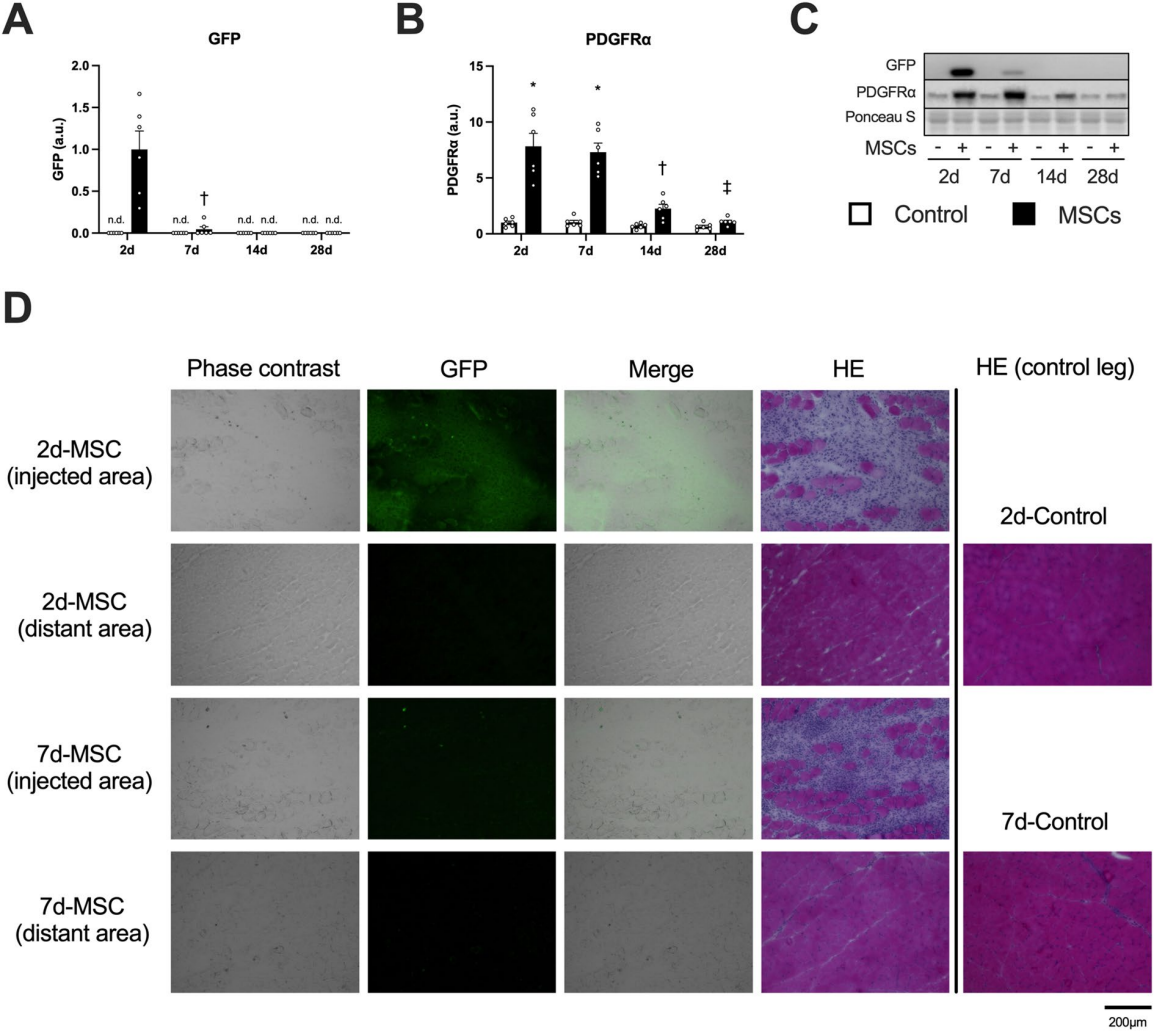
The expression of markers of MSCs after intramuscular injection. Protein expression of GFP (**A**), PDGFRα (**B**), representative bands (**C**), and cross-sections showing a phase-contrast image, localization of GFP, and stained with hematoxylin & eosin (**D**). Data are expressed relative to the injected leg (**A**) or control leg (**B**) 2 days post intramuscular injection of MSCs as the mean ± SE. ^*^P < 0.05 vs Control, ^†^P < 0.05 vs ipsilateral muscle at 2 days post-injection, and ^‡^P < 0.05 vs ipsilateral muscle at 7 days post-injection.

We detected GFP only in muscles injected with MSCs at 2 and 7 days after injection (Fig. 1A; Fig. 1C indicates representative blot data). The expression of platelet-derived growth factor receptor alpha (PDGFRα), a surface marker of MSC, increased at 2 and 7 days after the injection of MSCs (Fig. 1B). At the injected region, GFP-positive cells were observed at 2 days after the injection of MSCs but were significantly decreased at 7 days after the injection. Moreover, some muscle fibers showing swollen, round shape, and the sarcoplasm stained faintly, and small fibers having centrally located nucleus were observed by hematoxylin and eosin staining at 2 and 7 days after the injection (Fig. 1D). The injection of MSCs increased wet muscle weight at 2 days after the injection but returned to control levels at 7 days after the injection (Table 2). Based on these results, we analyzed data at 2 and 7 days after the injection when the MSCs remained within the muscle tissue.

**Table 2:** Body and muscle weight.

	Body weight (g)	Muscle wet weight (mg)		Muscle wet weight/body weight (mg/g)	
		Control	MSC	Control	MSC
2d	25.3 ± 0.6	131.7 ± 4.4	157.0 ± 3.4*	5.2 ± 0.2	6.2 ± 0.2*
7d	25.0 ± 0.6	134.0 ± 4.2	138.7 ± 3.9^†^	5.4 ± 0.1	5.5 ± 0.1^†^
14d	26.1 ± 0.4	135.9 ± 2.9	139.0 ± 1.6^†^	5.2 ± 0.1	5.3 ± 0.1^†^
28d	26.5 ± 1.2	143.9 ± 6.4	146.3 ± 6.0	5.4 ± 0.1	5.5 ± 0.1^†^

*P < 0.05 vs Control and ^†^P < 0.05 vs ipsilateral muscle at 2 days post-injection.

### Muscle satellite cell-related factors

**Figure 2:**
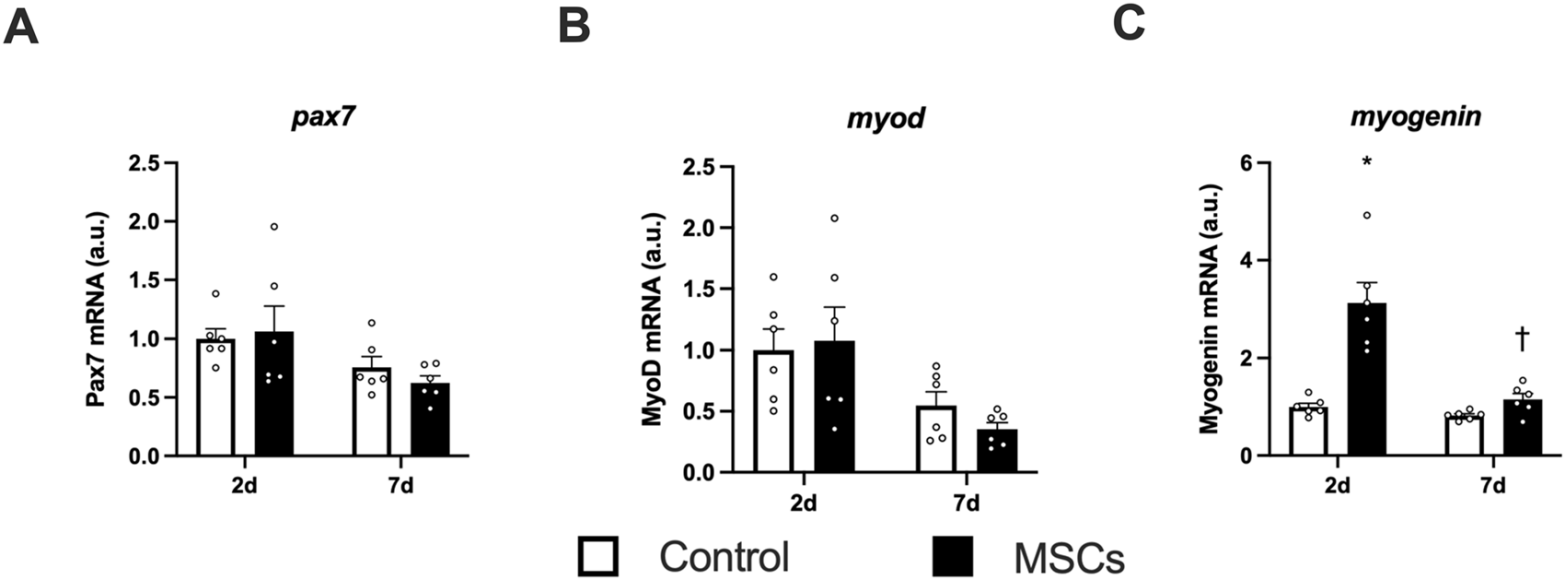
mRNA expression of muscle satellite cell-related markers. Expression of genes encoding Pax7 (**A**), MyoD (**B**), and Myogenin (**C**). Data are expressed relative to the control leg at 2 days post intramuscular injection of MSCs as the mean ± SE. A significant main effect of time was observed in MyoD (P < 0.05). ^*^P < 0.05 vs Control and ^†^P < 0.05 vs ipsilateral muscle at 2 days post-injection.

The mRNA expression of Pax7 did not change after the injection of MSCs (Fig. 2A). The mRNA expression of MyoD decreased in both sides of muscle at 7 days after the injection (main effect of time, Fig. 2B). The mRNA expression of myogenin increased at 2 days after the injection of MSCs (Fig. 2C).

### mTORC1-related factors and muscle protein synthesis

**Figure 3:**
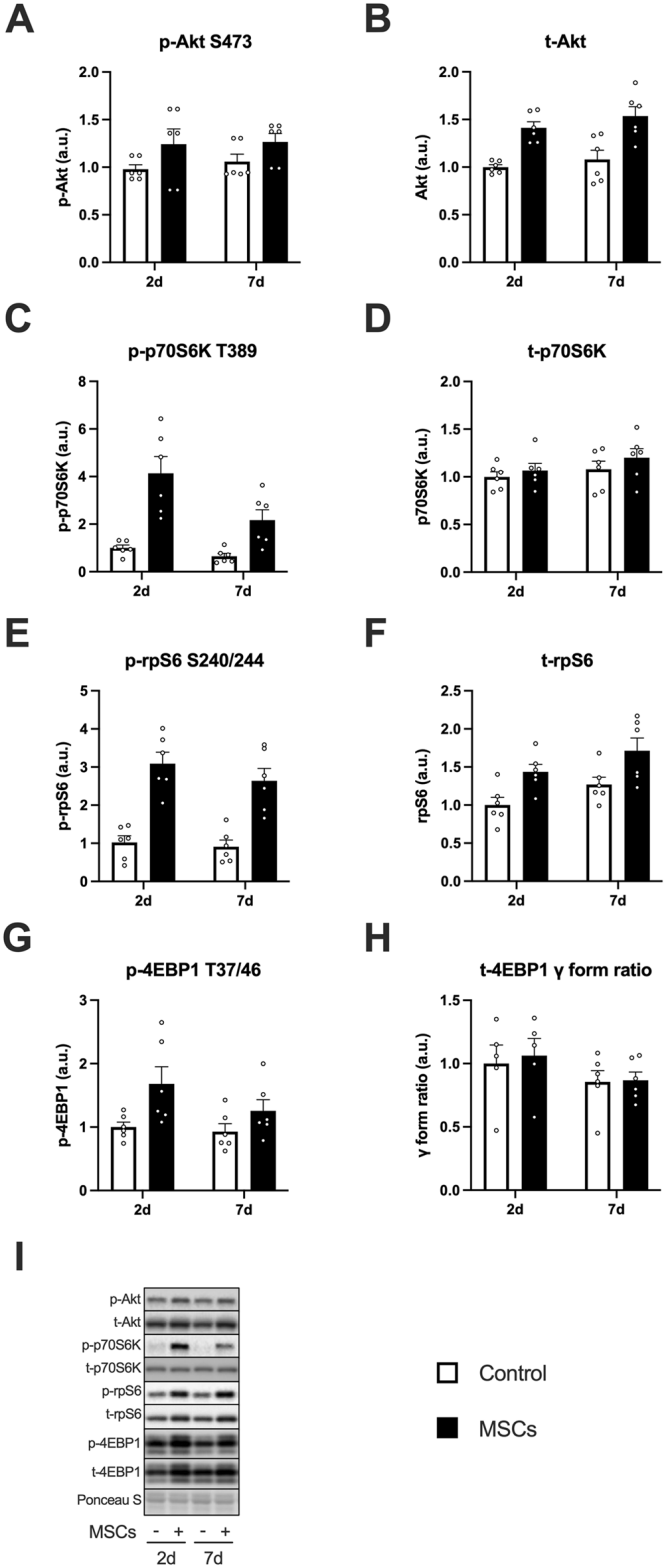
The expression of mTORC1 signal-related factors after intramuscular injection of MSCs. Protein expression of phosphorylated Akt (Ser473, **A**), Akt (**B**), phosphorylated p70S6K (Thr389, **C**), p70S6K (**D**), phosphorylated rpS6 (Ser240/244, **E**), rpS6 (**F**), phosphorylated 4EBP1 (Thr37/46, **G**), 4EBP1 gamma form ratio (**H**), and representative bands (**I**). Data are expressed relative to the control leg at 2 days post intramuscular injection of MSCs as the mean ± SE. Tendency or significant main effects of MSC were observed for phosphorylated Akt, Akt, phosphorylated p70S6K, p70S6K, phosphorylated rpS6, rpS6, and phosphorylated 4EBP1 (P = 0.0581, P < 0.0001, P < 0.001, P < 0.01, P < 0.0001, P < 0.001, and P < 0.001, respectively). A significant main effect of time was observed for phosphorylated p70S6K (P < 0.05).

**Figure 4:**
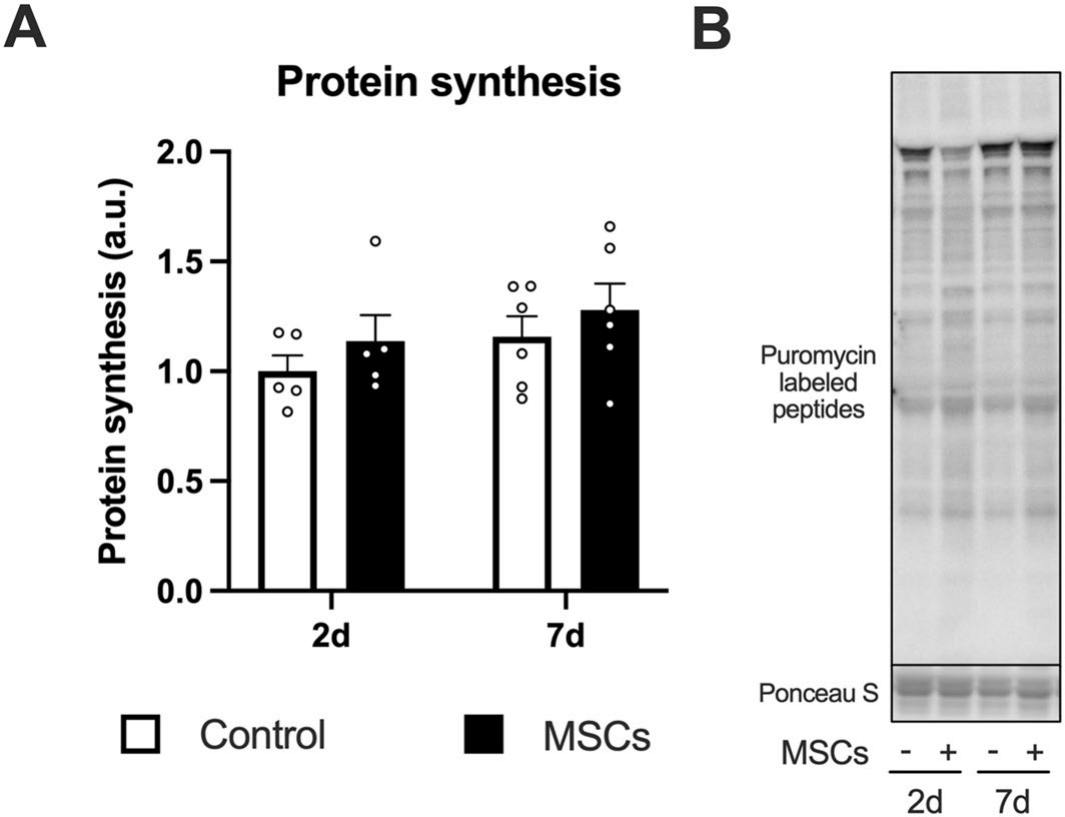
Muscle protein synthesis after intramuscular injection of MSCs. Data are expressed relative to the control leg at 2 days post intramuscular injection of MSCs as the mean ± SE. A significant main effect of MSC was observed (P < 0.05).

The expression of phosphorylated Akt (at Ser473) and total Akt increased after the injection of MSCs (the main effect of MSCs, Fig. 3A,B). The expression of phosphorylated p70S6K (at Thr389) increased after the injection of MSCs (main effect of MSCs; Fig. 3I indicates representative blot data), and the magnitude decreased over time (main effect of time, Fig. 3C). We observed a slight increase in total p70S6K expression after the injection of MSCs (main effect of MSCs) but did not observe the main effect of time or the interaction (Fig. 3D). The expression of phosphorylated rpS6 (at Ser240/244) and total rpS6 increased after the injection of MSCs (main effect of MSCs, Fig. 3E,F). We observed similar results (i.e., significant MSC effect but no time effect or interaction) in phosphorylated 4EBP1 (at Thr37/46, Fig. 3G). The gamma form ratio of total 4EBP1 did not change after the injection of MSCs (Fig. 3H). Muscle protein synthesis increased after the injection of MSCs (main effect of MSCs), but no time effect or interaction was observed (Fig. 4A,B indicates representative blot data).

### Muscle protein breakdown related factors

**Figure 5:**
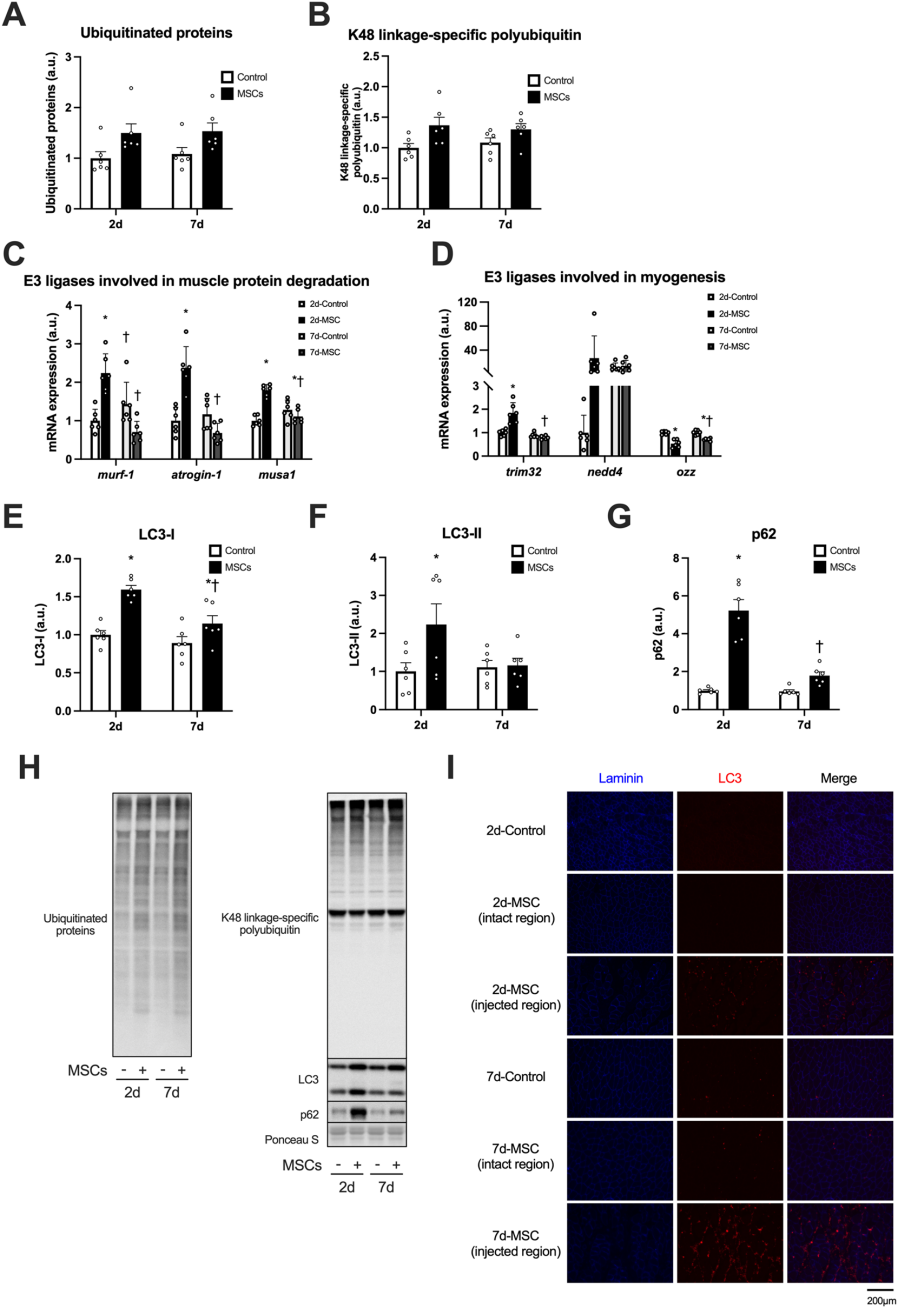
The expression of muscle catabolic systems-related factors after intramuscular injection of MSCs. Protein expression of ubiquitinated proteins (**A**) and K48 linkage-specific polyubiquitin (**B**), mRNA expression of E3 ligases involved in muscle protein degradation (MuRF-1, Atrogin-1, and MUSA1, **C**), and involved in myogenesis (TRIM32, Nedd4, and Ozz, **D**), protein expression of LC3-I (**E**), LC3-II (**F**), and p62 (**G**), representative bands (**H**), and cross-sections of muscle showing localization of laminin and LC3 (**I**). Data are expressed relative to the control leg at 2 days post intramuscular injection of MSCs as the mean ± SE. Significant main effects of MSC were observed in ubiquitinated proteins and K48 linkage-specific polyubiquitin (P < 0.0001 in both). ^*^P < 0.05 vs Control and ^†^P < 0.05 vs ipsilateral muscle at 2 days post-injection.

The expression of ubiquitinated proteins increased after the injection of MSCs (the main effect of MSCs, Fig. 5A,H indicates representative blot data). The expression of K48 linkage-specific polyubiquitin also increased after the injection of MSCs (main effect of MSCs, Fig. 5B). The mRNA expression of MuRF-1, a muscle-specific E3 ubiquitin ligase, increased at 2 days after the injection of MSCs (Fig. 5C). Similarly, the mRNA expression of atrogin-1, another muscle-specific E3 ubiquitin ligase, increased 2 days after the injection (Fig. 5C). Another muscle-specific E3 ubiquitin ligase MUSA1 increased at 2 days but decreased at 7 days after the injection of MSCs (Fig. 5C). The mRNA expression of TRIM32, one of the E3 ubiquitin ligases involved in myogenesis, increased 2 days after the injection (Fig. 5D). The mRNA expression of Nedd4 did not change significantly (Fig. 5D). The mRNA expression of Ozz, another ubiquitin ligase involved in myogenesis, decreased with the injection of MSCs, but slightly recovered at 7 days after the injection (Fig. 5D). The expression of LC3-I increased at 2 and 7 days after the injection of MSCs (Fig. 5E). The expression of LC3-II increased only at 2 days after the injection of MSCs (Fig. 5F). Similarly, expression of p62/SQSTM increased only at 2 days after the injection of MSCs (Fig. 5G). Finally, the expression of LC3 was primarily observed in the interstitial space of the injected region (Fig. 5I).

### IGF-1 and ERK1/2

**Figure 6:**
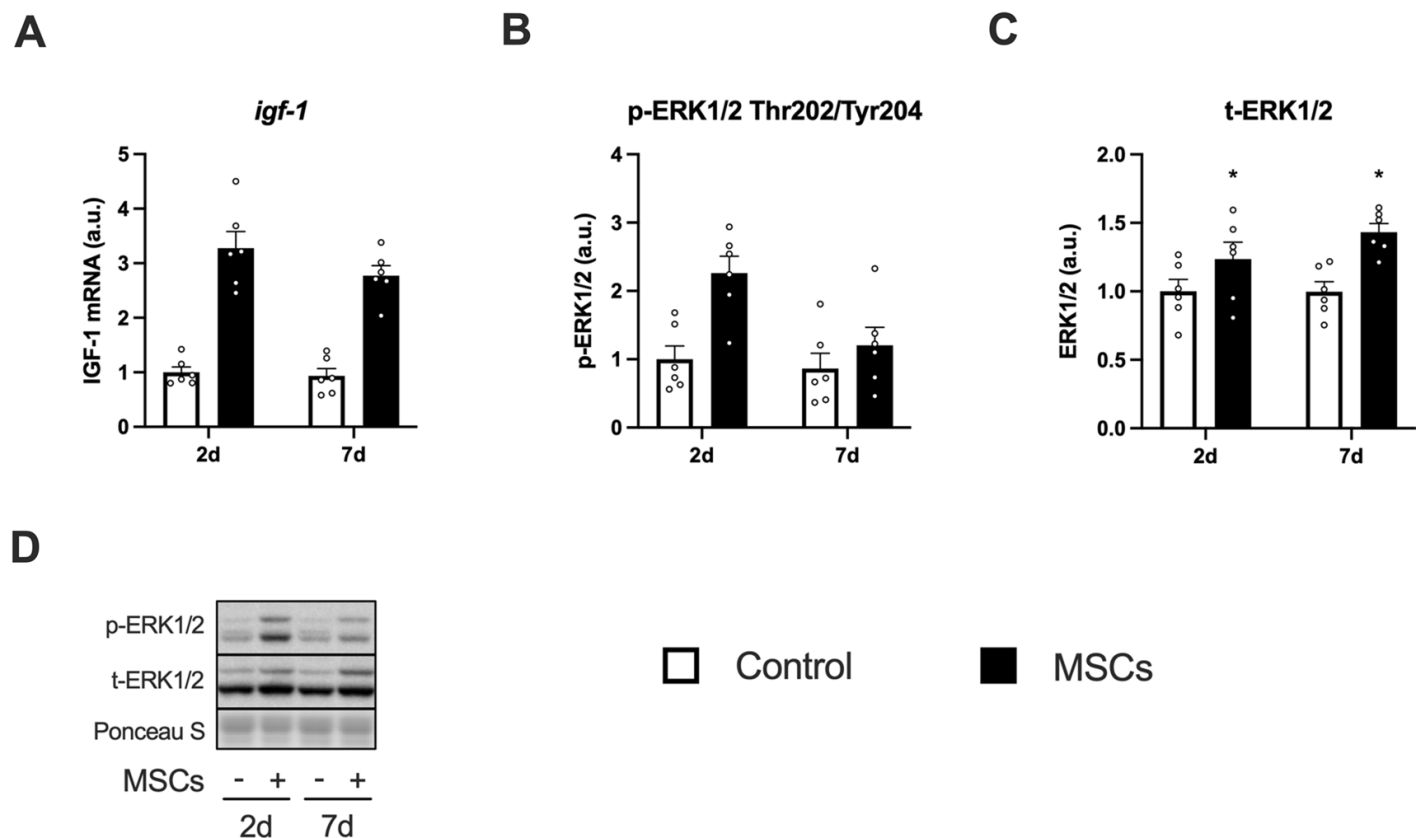
The expression of IGF-1 and ERK1/2 after intramuscular injection of MSCs. Expression of a gene encoding IGF-1 (**A**), protein expression of phosphorylated ERK1/2 (Thr202/Tyr204, **B**), ERK1/2 (**C**), and representative bands (**D**). Data are expressed relative to the control leg at 2 days post intramuscular injection of MSCs as the mean ± SE. Significant main effects of MSC were observed in IGF-1 and phosphorylated ERK1/2 (P < 0.0001 and P < 0.01, respectively). A significant main effect of time was observed in phosphorylated ERK1/2 (P < 0.05). ^*^P < 0.05 vs Control and ^†^P < 0.05 vs ipsilateral muscle at 2 days post-injection.

The mRNA expression of IGF-1 increased at 2 and 7 days after the injection of MSCs (Fig. 6A). The expression of phosphorylated ERK1/2 increased after the injection of MSCs (main effect of MSCs), and the magnitude decreased over time (main effect of time, Fig. 6B,D indicates representative blot data). The expression of total ERK1/2 was increased at both 2 and 7 days after the injection of MSCs (Fig. 6C).

## Discussion

The main findings of the study are as follows: (1) markers of MSCs remained within the muscle tissue for 7 days after intramuscular injection; (2) intramuscular injection of MSCs activated transcription of differentiation marker of muscle satellite cells; (3) intramuscular injection of MSCs activated muscle anabolic and catabolic signals; and (4) intramuscular injection of MSCs increased transcription of IGF-1. These results suggest that intramuscular injection of MSCs accelerates skeletal muscle protein turnover.

We observed that intramuscularly-injected MSCs remained in the muscle for 7 days, indicated by GFP detection and increased expression of PDGFRα. Nakabayashi et al. reported that MSCs transplanted into injured skeletal muscle (1.0 × 10^6^ cells into the anterior tibialis muscle) decreased within 24 h after transplantation in rats^36^. Oshima et al. reported that MSCs transplanted into injured skeletal muscle (1.0 × 10^5^ cells into the anterior tibialis muscle) were not detected 1 week after injection in rats^24^. In the present study, we intramuscularly injected a higher number of MSCs (2.0 × 10^6^ cells), which might be responsible for longer retention times. However, we failed to detect MSCs in the muscle 14 and 28 days after injection. A previous study reported that intramuscularly injected MSCs (4.0 × 10^5^ cells into the gastrocnemius muscle) 1 h after a single bout of downhill exercise remained for 2 weeks^30^. These findings suggest that the MSCs may require additional interventions to remain within the skeletal muscle tissue for extended periods.

Although the previous studies reported that simple injection of MSCs did not increase Pax7-positive nuclei in skeletal muscle^30,36^, the injection of MSCs evoked activation of satellite cells indicated by an increase of myogenin mRNA expression in the present study. In the previous studies, the tissue was analyzed more than 1 week after the injection^30,36^. In the current study, the changes in mRNA expressions of myogenin were observed within 2 or 7 days after the injection, suggesting that the increased mRNA expressions may occur in the early phase after the injection. It remains unclear whether this increase was caused by differentiation of the MSCs into satellite cells or activation of native satellite cells in the skeletal muscle. Elucidating these points would be essential to develop the application of MSCs to support skeletal muscle regeneration or hypertrophy.

The ubiquitin–proteasome system and the autophagy-lysosome system are two proteolytic systems that mediate protein degradation in the skeletal muscle. Ubiquitinated proteins and autophagosomes are degraded by 26S proteasomes and lysosomes, respectively^18,37^. In the present study, the injection of MSCs increased ubiquitinated proteins and K48-linkage-specific polyubiquitin. Increased transcription of MuRF-1, atrogin-1, and MUSA1, specific E3 ubiquitin ligases involved in skeletal muscle protein degradation, was observed^37,38^. The injection of MSCs affected transcription of TRIM 32 and Ozz, which are involved in myogenesis and myofiber differentiation^39–41^. Considering no change in mRNA expression of Pax7, the injection of MSCs activated protein anabolism and protein catabolism, and the latter was likely through the ubiquitin–proteasome system and muscle degeneration using the existing muscle stem cells. On the other hand, increased expression of LC3-II (an indicator of autophagosome formation)^42^ was observed; however, the LC3 was primarily distributed in interstitial spaces. These results suggest that the injection of MSCs activated both the ubiquitin–proteasome system and autophagy, while the latter likely did not primarily affect muscle fiber. A previous study reported that macrophages eliminated MSCs injected into regenerating tissues, including skeletal muscle^43^. Autophagy was reported to be activated in macrophages by stimulation of inflammatory cytokines^44^. These facts and morphological observation led us to speculate that the macrophages eliminated the injected MSCs in the present study. From the viewpoint of maintaining a positive balance of protein metabolism, reducing the activation of protein catabolic systems in the skeletal muscle would be essential. Park et al. reported that autophagy was essential for the differentiation of the MSCs into myocytes^45^. These facts suggest that activating protein catabolic systems in the skeletal muscle injected with the MSCs might be necessary for skeletal muscle tissue growth. To evaluate the authentic role of the upregulation of protein catabolic systems, especially autophagy, after injection of the MSCs and to develop the methods for application of MSCs for skeletal muscle tissue, further studies with detailed histological and related analysis are required.

p70S6K and 4EBP1 are the primary downstream targets of the mTORC1 pathway, and rpS6 is the downstream target of p70S6K. In the present study, the injection of MSCs increased phosphorylation of these factors, and the duration of the mTORC1 activation corresponded to the presence of the MSCs in the skeletal muscle. These findings suggest that the injection of MSCs activated mTORC1. A previous study reported that four times the injection of MSCs (1.0 × 10^6^, once per every week) over 4 weeks increased phosphorylation of p70S6K^Thr389^ in mdx mouse skeletal muscle, which is in line with our findings^46^. Furthermore, the injection of MSCs also augmented muscle protein synthesis. These findings suggest that the intramuscular injection of MSCs activates mTORC1 signaling and muscle protein synthesis in skeletal muscle.

We found that the injection of MSCs increased the transcription of IGF-1 in the skeletal muscle. The phosphorylation of ERK1/2^Thr202^^/Tyr204^, one of the downstream targets of IGF-1^47,48^, was increased by the injection of MSCs. These findings lead us to believe that the intramuscular injection of MSCs would increase the secretion of IGF-1 in the skeletal muscle as hypothesized, which activated mTORC1 and protein synthesis. MSCs secrete growth factors, including IGF-1, fibroblast growth factor (FGF) 2, and vascular endothelial growth factor (VEGF)^27,49^. Although IGF-1 is known to stimulate muscle protein anabolism^12,50^, other growth factors are also involved in the activation of mTORC1. Shi et al. reported that the subcutaneous injection of FGF2 enhanced phosphorylation of Akt and mTOR in injured rat skeletal muscle^51^. Huey et al. reported that skeletal muscle-specific VEGF knockout mice showed an increase in IGF-1 expression but did not increase phosphorylation of Akt in functionally overloaded plantaris muscle, suggesting that VEGF stimulates Akt with IGF-1 synergistically^52^. These findings suggest that other growth factors secreted by the injected MSCs may have also contributed to our observation of enhanced mTORC1 activation. Elucidating the contribution of each factor on mTORC1 and muscle protein synthesis would strengthen the evidence supporting the application of MSCs and provide insight into other ways of applying MSCs.

The present study has some limitations. First, we did not clarify how MSCs stimulated the observed effect. As there were no GFP-positive muscles fiber 7 days after the injection, the injected MSCs were not likely to differentiate into muscle fibers. However, as there were GFP-positive cells in the injected area, the possibility of a paracrine effect by MSCs cannot be ruled out. On the other hand, a previous study reported that the intra-cardiac injection of adult stem cells after cardiac ischemic injury showed therapeutic effect through acute immune response^53^. Skeletal muscle injury causes accumulation of macrophages, activation of satellite cells, and paracrine of IGF-1, which overlap the observations in the present study^54^. These facts and the results from morphological analysis led us to consider that the immune response, muscle damage, and regeneration process might have caused the effect observed in the present study. Second, we did not provide a placebo group injected with vehicle or equal-sized particles. Because MSCs tend to accumulate at sites of inflammation^55^, leaked MSCs from the injected muscle might accumulate in the contralateral muscle injected with the vehicle or placebo. Considering this point and the ethical aspects, we left the control leg intact. Ideally, a placebo group injected with particles of equal size to MSCs should be provided.

In conclusion, intramuscularly-injected MSCs remained in the injected muscle for 7 days. Although the injection of MSCs also increased protein ubiquitination and autophagosome formation, MSC injection activated the transcription of differentiation markers of muscle satellite cells, mTORC1 signaling, muscle protein synthesis, and transcription of IGF-1, suggesting augmented muscle protein turnover.

## Supplementary Information

Supplementary Information 1.

Supplementary Information 2.

## References

[1] Landi F (2012). Sarcopenia as a risk factor for falls in elderly individuals: Results from the ilSIRENTE study. Clin. Nutr..

[2] Mesinovic J, Zengin A, De Courten B, Ebeling PR, Scott D (2019). Sarcopenia and type 2 diabetes mellitus: A bidirectional relationship. Diabetes. Metab. Syndr. Obes..

[3] Atkins JL (2014). Sarcopenic obesity and risk of cardiovascular disease and mortality: A population-based cohort study of older men. J. Am. Geriatr. Soc..

[4] Denison HJ, Cooper C, Sayer AA, Robinson SM (2015). Prevention and optimal management of sarcopenia: A review of combined exercise and nutrition interventions to improve muscle outcomes in older people. Clin. Interv. Aging..

[5] Haran PH, Rivas DA, Fielding RA (2012). Role and potential mechanisms of anabolic resistance in sarcopenia. J. Cachexia. Sarcopenia. Muscle..

[6] Phillips SM, Tipton KD, Aarsland A, Wolf SE, Wolfe RR (1997). Mixed muscle protein synthesis and breakdown after resistance exercise in humans. Am. J. Physiol..

[7] Drummond MJ, Rasmussen BB (2008). Leucine-enriched nutrients and the regulation of mammalian target of rapamycin signalling and human skeletal muscle protein synthesis. Curr. Opin. Clin. Nutr. Metab. Care..

[8] Schiaffino S, Dyar KA, Ciciliot S, Blaauw B, Sandri M (2013). Mechanisms regulating skeletal muscle growth and atrophy. FEBS. J..

[9] Dreyer HC (2006). Resistance exercise increases AMPK activity and reduces 4E-BP1 phosphorylation and protein synthesis in human skeletal muscle. J. Physiol..

[10] Ogasawara R, Sato K, Matsutani K, Nakazato K, Fujita S (2014). The order of concurrent endurance and resistance exercise modifies mTOR signaling and protein synthesis in rat skeletal muscle. Am. J. Physiol. Endocrinol. Metab..

[11] Dickinson JM (2011). Mammalian target of rapamycin complex 1 activation is required for the stimulation of human skeletal muscle protein synthesis by essential amino acids. J. Nutr..

[12] Rommel C (2001). Mediation of IGF-1-induced skeletal myotube hypertrophy by PI(3)K/Akt/mTOR and PI(3)K/Akt/GSK3 pathways. Nat. Cell. Biol..

[13] Baar K, Esser K (1999). Phosphorylation of p70S6kcorrelates with increased skeletal muscle mass following resistance exercise. Am. J. Physiol. Cell. Physiol..

[14] You J-S (2018). The role of raptor in the mechanical load-induced regulation of mTOR signaling, protein synthesis, and skeletal muscle hypertrophy. FASEB J..

[15] Clarke BA (2007). The E3 Ligase MuRF1 degrades myosin heavy chain protein in dexamethasone-treated skeletal muscle. Cell. Metab..

[16] Cohen S (2009). During muscle atrophy, thick, but not thin, filament components are degraded by MuRF1-dependent ubiquitylation. J. Cell. Biol..

[17] Polge C (2011). Muscle actin is polyubiquitinylated in vitro and in vivo and targeted for breakdown by the E3 ligase MuRF1. FASEB. J..

[18] Green DR, Levine B (2014). To be or not to be? How selective autophagy and cell death govern cell fate. Cell.

[19] Sandri M (2004). Foxo transcription factors induce the atrophy-related ubiquitin ligase atrogin-1 and cause skeletal muscle atrophy. Cell.

[20] Chan EY (2009). mTORC1 phosphorylates the ULK1-mAtg13-FIP200 autophagy regulatory complex. Sci. Signal..

[21] Dezawa M (2005). Bone marrow stromal cells generate muscle cells and repair muscle degeneration. Science.

[22] Linard C (2018). Long-term effectiveness of local BM-MSCs for skeletal muscle regeneration: A proof of concept obtained on a pig model of severe radiation burn. Stem. Cell. Res. Ther..

[23] Ninagawa NT (2013). Transplantated mesenchymal stem cells derived from embryonic stem cells promote muscle regeneration and accelerate functional recovery of injured skeletal muscle. Biores. Open. Access..

[24] Oshima S, Kamei N, Nakasa T, Yasunaga Y, Ochi M (2014). Enhancement of muscle repair using human mesenchymal stem cells with a magnetic targeting system in a subchronic muscle injury model. J. Orthopc. Sci..

[25] Winkler T (2008). Dose–response relationship of mesenchymal stem cell transplantation and functional regeneration after severe skeletal muscle injury in rats. Tissue. Eng. Part. A..

[26] Ranganath SH, Levy O, Inamdar MS, Karp JM (2012). Harnessing the mesenchymal stem cell secretome for the treatment of cardiovascular disease. Cell Stem Cell.

[27] Kinnaird T (2004). Local delivery of marrow-derived stromal cells augments collateral perfusion through paracrine mechanisms. Circulation.

[28] Santa María L, Rojas CV, Minguell JJ (2004). Signals from damaged but not undamaged skeletal muscle induce myogenic differentiation of rat bone-marrow-derived mesenchymal stem cells. Exp. Cell. Res..

[29] Witt R (2017). Mesenchymal stem cells and myoblast differentiation under HGF and IGF-1 stimulation for 3D skeletal muscle tissue engineering. BMC. Cell. Biol..

[30] Zou K (2015). Mesenchymal stem cells augment the adaptive response to eccentric exercise. Med. Sci. Sport. Exerc..

[31] Abrigo J (2016). High fat diet-induced skeletal muscle wasting is decreased by mesenchymal stem cells administration: Implications on oxidative stress, ubiquitin proteasome pathway activation, and myonuclear apoptosis. Oxid. Med. Cell. Longev..

[32] Crisostomo PR (2008). Human mesenchymal stem cells stimulated by TNF-α, LPS, or hypoxia produce growth factors by an NFκB-but not JNK-dependent mechanism. Am. J. Physiol. Cell. Physiol..

[33] Goodman CA (2011). Novel insights into the regulation of skeletal muscle protein synthesis as revealed by a new nonradioactive in vivo technique. FASEB. J..

[34] Takegaki J, Sase K, Fujita S (2019). Repeated bouts of resistance exercise attenuate mitogen-activated protein-kinase signal responses in rat skeletal muscle. Biochem. Biophys. Res. Commun..

[35] Takegaki J (2019). Influence of shortened recovery between resistance exercise sessions on muscle-hypertrophic effect in rat skeletal muscle. Physiol. Rep..

[36] Nakabayashi A (2013). In vivo bioluminescence imaging of magnetically targeted bone marrow-derived mesenchymal stem cells in skeletal muscle injury model. J. Orthop. Res..

[37] Bodine SC, Baehr LM (2014). Skeletal muscle atrophy and the E3 ubiquitin ligases MuRF1 and MAFbx/atrogin-1. Am. J. Physiol. Endocrinol. Metab..

[38] Sartori R (2013). BMP signaling controls muscle mass. Nat. Genet..

[39] Campos Y (2010). Ozz-E3 ubiquitin ligase targets sarcomeric embryonic myosin heavy chain during muscle development. PLoS One.

[40] Nastasi T (2004). Ozz-E3, a muscle-specific ubiquitin ligase, regulates β-Catenin degradation during myogenesis. Dev. Cell.

[41] Nicklas S (2012). TRIM32 regulates skeletal muscle stem cell differentiation and is necessary for normal adult muscle regeneration. PLoS One.

[42] Mizushima N, Yoshimori T (2007). How to interpret LC3 immunoblotting. Autophagy.

[43] Arutyunyan I (2015). Elimination of allogeneic multipotent stromal cells by host macrophages in different models of regeneration. Int. J. Clin. Exp. Pathol..

[44] Xu Y (2007). Toll-like receptor 4 is a sensor for autophagy associated with innate immunity. Immunity.

[45] Park S (2017). Autophagy induction in the skeletal myogenic differentiation of human tonsil-derived mesenchymal stem cells. Int. J. Mol. Med..

[46] Pinheiro CH (2012). Local injections of adipose-derived mesenchymal stem cells modulate inflammation and increase angiogenesis ameliorating the dystrophic phenotype in dystrophin-deficient skeletal muscle. Stem. Cell. Rev. Rep..

[47] Haddad F, Adams GR (2004). Inhibition of MAP/ERK kinase prevents IGF-I-induced hypertrophy in rat muscles. J. Appl. Physiol..

[48] Adams GR (2002). Invited review: Autocrine/paracrine IGF-I and skeletal muscle adaptation. J. Appl. Physiol..

[49] Kinnaird T (2004). Marrow-derived stromal cells express genes encoding a broad spectrum of arteriogenic cytokines and promote in vitro and in vivo arteriogenesis through paracrine mechanisms. Circ. Res..

[50] Bark TH, McNurlan MA, Lang CH, Garlick PJ (1998). Increased protein synthesis after acute IGF-I or insulin infusion is localized to muscle in mice. Am. J. Physiol. Endocrinol. Metab..

[51] Shi H (2016). Myoprotective effects of bFGF on skeletal muscle injury in pressure-related deep tissue injury in rats. Burns. Trauma..

[52] Huey KA, Smith SA, Sulaeman A, Breen EC (2016). Skeletal myofiber VEGF is necessary for myogenic and contractile adaptations to functional overload of the plantaris in adult mice. J. Appl. Physiol..

[53] Vagnozzi RJ (2019). An acute immune response underlies the benefit of cardiac stem-cell therapy. Nature.

[54] Tonkin J (2015). Monocyte/macrophage-derived IGF-1 orchestrates murine skeletal muscle regeneration and modulates autocrine polarization. Mol. Ther..

[55] Eggenhofer E, Luk F, Dahlke MH, Hoogduijn MJ (2014). The life and fate of mesenchymal stem cells. Front. Immunol..

