# Supplementary material for: Intramuscular injection of mesenchymal stem cells activates anabolic and catabolic systems in mouse skeletal muscle
Source: Sci Rep. 2021 Oct 27;11:21224. doi: 10.1038/s41598-021-00627-6 (PMC8551189; doi:10.1038/s41598-021-00627-6)
Supplement: Supplementary file 1 — Supplementary Information 1. [file 41598_2021_627_MOESM1_ESM.docx]

**Fig. S1. The effect of intramuscular injection of PBS.**

Twenty μL of phosphate buffered saline (PBS) was injected into the right gastrocnemius muscle of eleven-weeks old male C57BL/6J mice. The left gastrocnemius muscle was kept intact and served as the control. At 2, 7, 14, and 28 days after PBS injection, both gastrocnemius muscles were collected (n = 3 for each day). The collected muscle tissues were processed for western blotting analysis or SUnSET method as described in the methods section. Protein expression of platelet derived growth factor receptor alpha (PDGFRα, A), protein synthesis (B), phosphorylated p70S6K (Thr389, C), and ubiquitinated proteins (D). Data are expressed relative to the control leg at 2 days post intramuscular injection of PBS as the mean ±SE and analyzed using 2way ANOVA (PBS × time). If an interaction was observed, Bonferroni multiple-comparison testing was performed. *P < 0.05 vs Control.

**Fig. S2. The expression of housekeeping genes.**

The expression of 8 housekeeping genes (HPRT, GUS, CYC, GAPDH, ACTB, TBP, 18S, and B2M) in pool sample of each group were evaluated by qPCR. Data are expressed relative to control leg at 2 days post injection of MSCs as ⊿Ct. Primer sequences used for this analysis are shown in Table S1.

**Fig. S3. mRNA expression of genes shown in main data normalized by GAPDH.**

Expression of genes encoding Pax7 (A), MyoD (B), Myogenin (C), MuRF-1 (D), Atrogin-1 (E), MUSA1 (F), TRIM32 (G), Nedd4 (H), and Ozz (I) normalized by GAPDH. Data are expressed relative to the control leg at 2 days post intramuscular injection of MSCs as the mean ± SE. Significant main effects of MSC were observed in Nedd4 and Ozz (P < 0.01 in both). ^*^P < 0.05 vs Control and ^†^P < 0.05 vs ipsilateral muscle at 2 days post-injection.

**Table S1. Primer sequences of housekeeping genes for qPCR**

| Gene | Forward primer (5’-3’) | Reverse primer (5’-3’) |
| --- | --- | --- |
| *Hprt* | GCTTGCTGGTGAAAAGGACCTCTCGAAG | CCCTGAAGTACTCATTATAGTCAAGGGCAT |
| *Gus* | CACGGCGATGGACCCAAGAT | CCCATTCACCCACACAACTGC |
| *Cyc* | TCCGACTGTGGACAGCTCTA | ATTGCGAGCAGATGGGGTAG |
| *Gapdh* | CTCCCACTCTTCCACCTTCG | GCCTCTCTTGCTCAGTGTCC |
| *Actb* | GGCTGTATTCCCCTCCATCG | CCAGTTGGTAACAATGCCATGT |
| *Tbp* | TATGACCCCTATCACTCCTG | TTCTTCACTCTTGGCTCCTGT |
| *18s* | CCTGGATACCGCAGCTAGGA | GCGGCGCAATACGAATGCCCC |
| *B2m* | TTCTGGTGCTTGTCTCACTGA | CAGTATGTTCGGCTTCCCATTC |
