# Supplementary material for: Intramuscular injection of mesenchymal stem cells activates anabolic and catabolic systems in mouse skeletal muscle
Source: Sci Rep. 2021 Oct 27;11:21224. doi: 10.1038/s41598-021-00627-6 (PMC8551189; doi:10.1038/s41598-021-00627-6)
Supplement: Supplementary file 2 — Supplementary Information 2. [file 41598_2021_627_MOESM2_ESM.pdf]

Supplemental Information 1

Each representative bands for western blot analysis displayed in the figures were cropped from following parts (indicated by sideline). All bands are cropped from one place. Which sample loaded is indicated above. C: Common sample used for normalization between membrane, L: Left leg (Control), R: Right leg (MSC), and N: not included in the analysis. The image of Bright field were taken from same angle of view. The description below each ponceau S image shows the targets which the membrane used to detect.

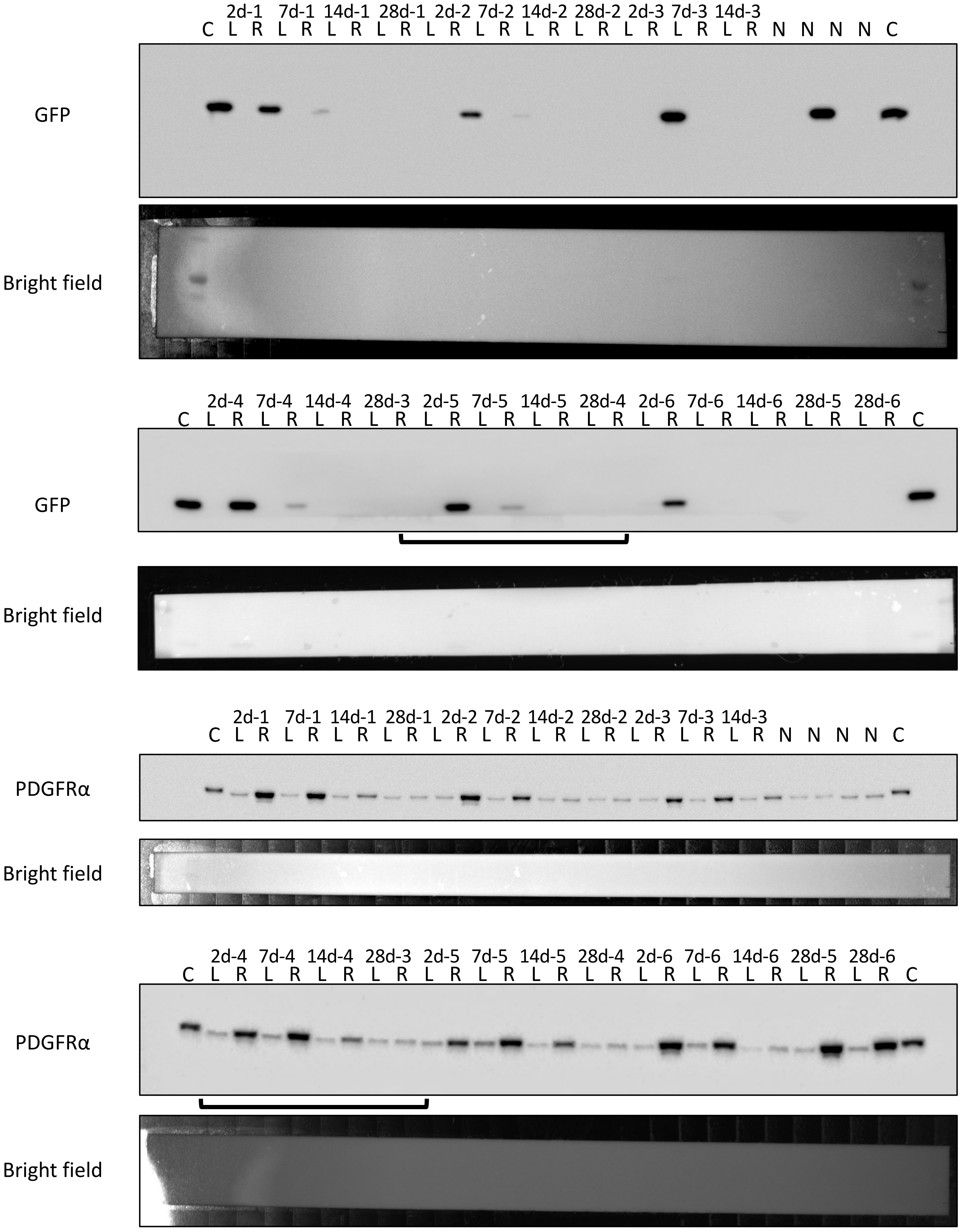

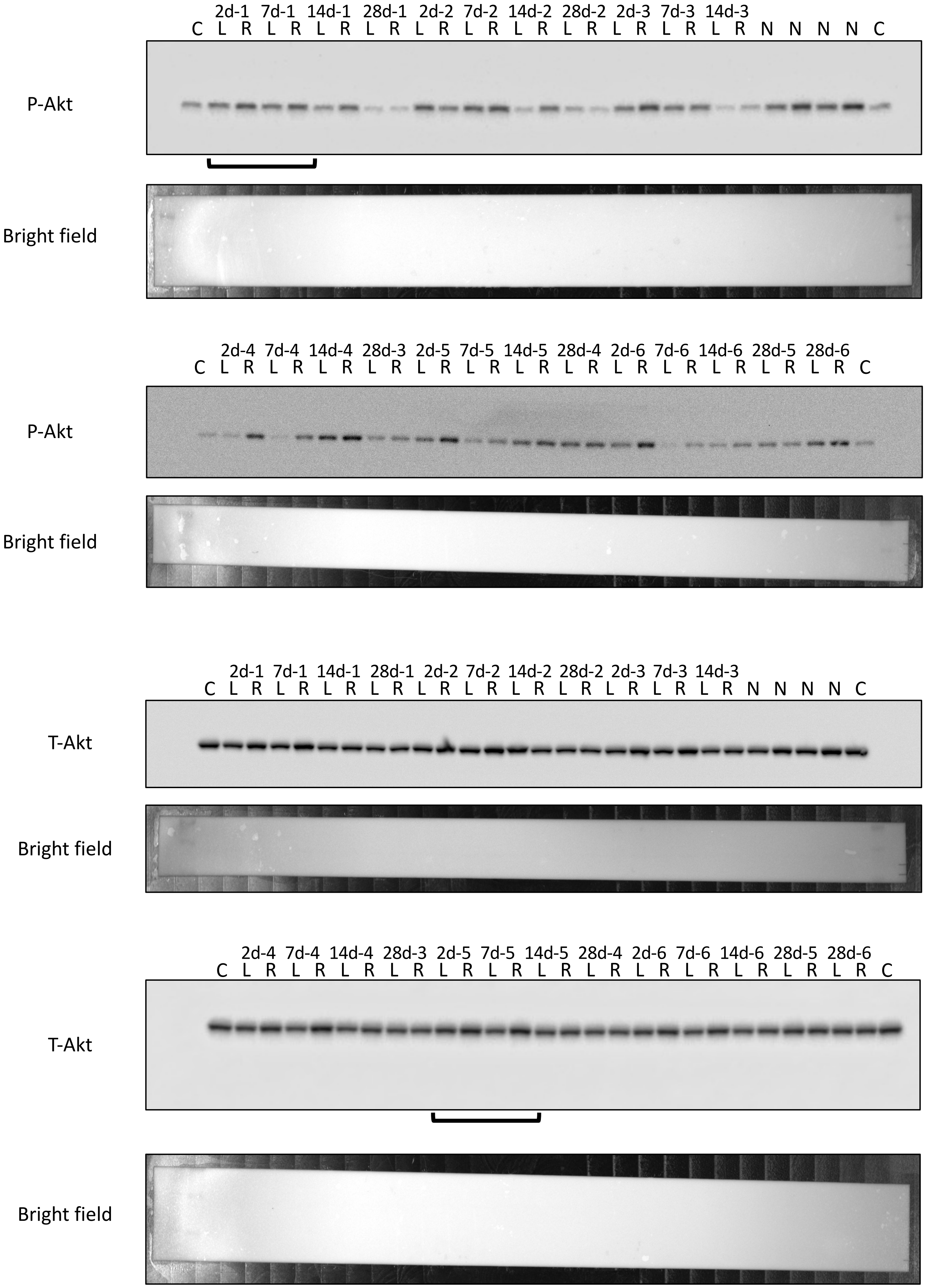
2d-1 7d-1 14d-1 28d-1 2d-2 7d-2 14d-2 28d-2 2d-3 7d-3 14d-3
C L R L R L R L R L R L R L R L R L R L R L R N N N N C
P-Akt
Bright field
2d-4 7d-4 14d-4 28d-3 2d-5 7d-5 14d-5 28d-4 2d-6 7d-6 14d-6 28d-5 28d-6
C L R L R L R L R L R L R L R L R L R L R L R L R L R C
P-Akt
Bright field
2d-1 7d-1 14d-1 28d-1 2d-2 7d-2 14d-2 28d-2 2d-3 7d-3 14d-3
C L R L R L R L R L R L R L R L R L R L R L R N N N N C
T-Akt
Bright field
2d-4 7d-4 14d-4 28d-3 2d-5 7d-5 14d-5 28d-4 2d-6 7d-6 14d-6 28d-5 28d-6
C L R L R L R L R L R L R L R L R L R L R L R L R L R C
T-Akt
Bright field

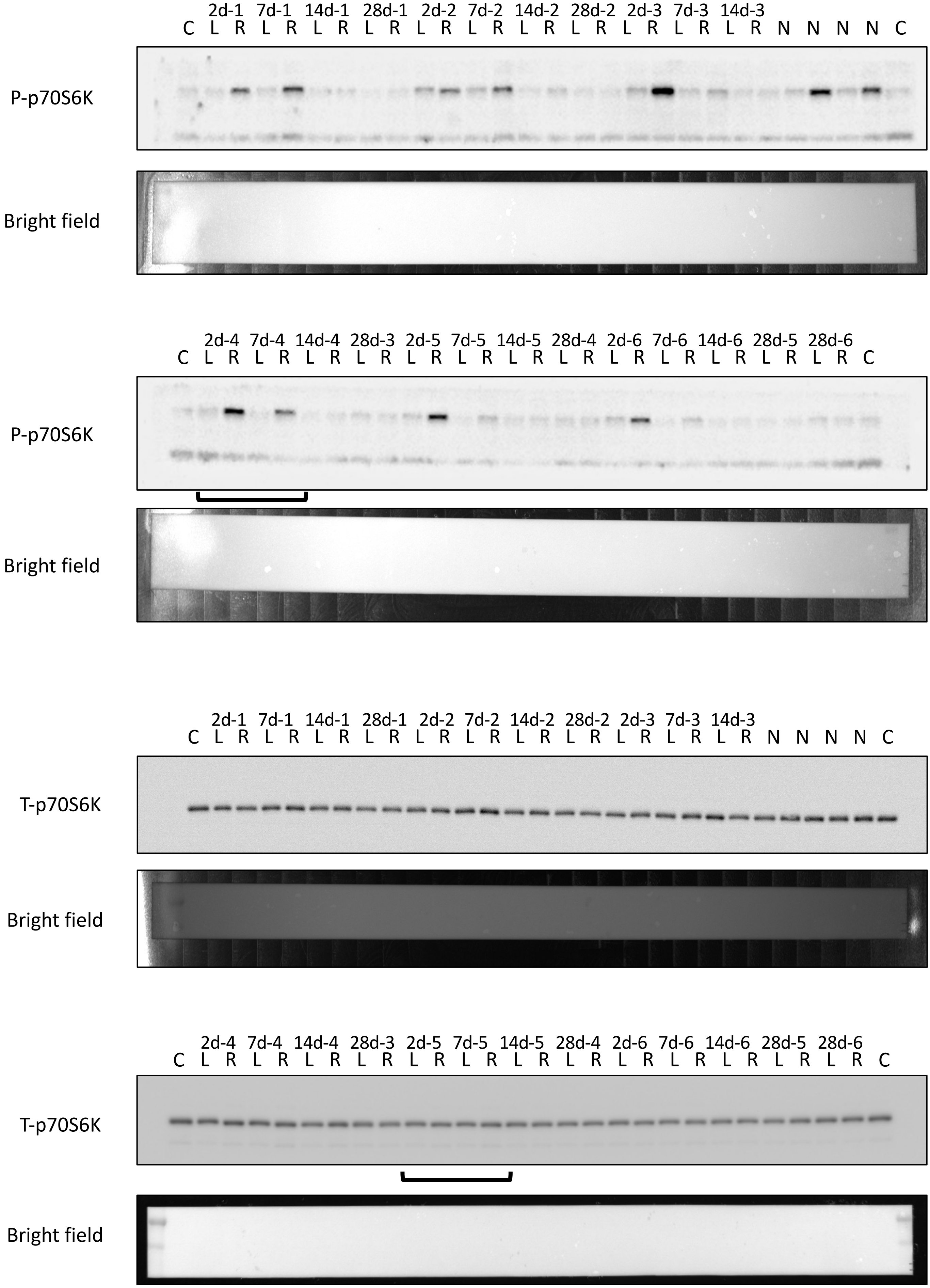
2d-1 7d-1 14d-1 28d-1 2d-2 7d-2 14d-2 28d-2 2d-3 7d-3 14d-3
C L R L R L R L R L R L R L R L R L R L R L R N N N N C
P-p70S6K
Bright field
2d-4 7d-4 14d-4 28d-3 2d-5 7d-5 14d-5 28d-4 2d-6 7d-6 14d-6 28d-5 28d-6
C L R L R L R L R L R L R L R L R L R L R L R L R L R C
P-p70S6K
Bright field
2d-1 7d-1 14d-1 28d-1 2d-2 7d-2 14d-2 28d-2 2d-3 7d-3 14d-3
C L R L R L R L R L R L R L R L R L R L R L R N N N N C
T-p70S6K
Bright field
2d-4 7d-4 14d-4 28d-3 2d-5 7d-5 14d-5 28d-4 2d-6 7d-6 14d-6 28d-5 28d-6
C L R L R L R L R L R L R L R L R L R L R L R L R L R C
T-p70S6K
Bright field

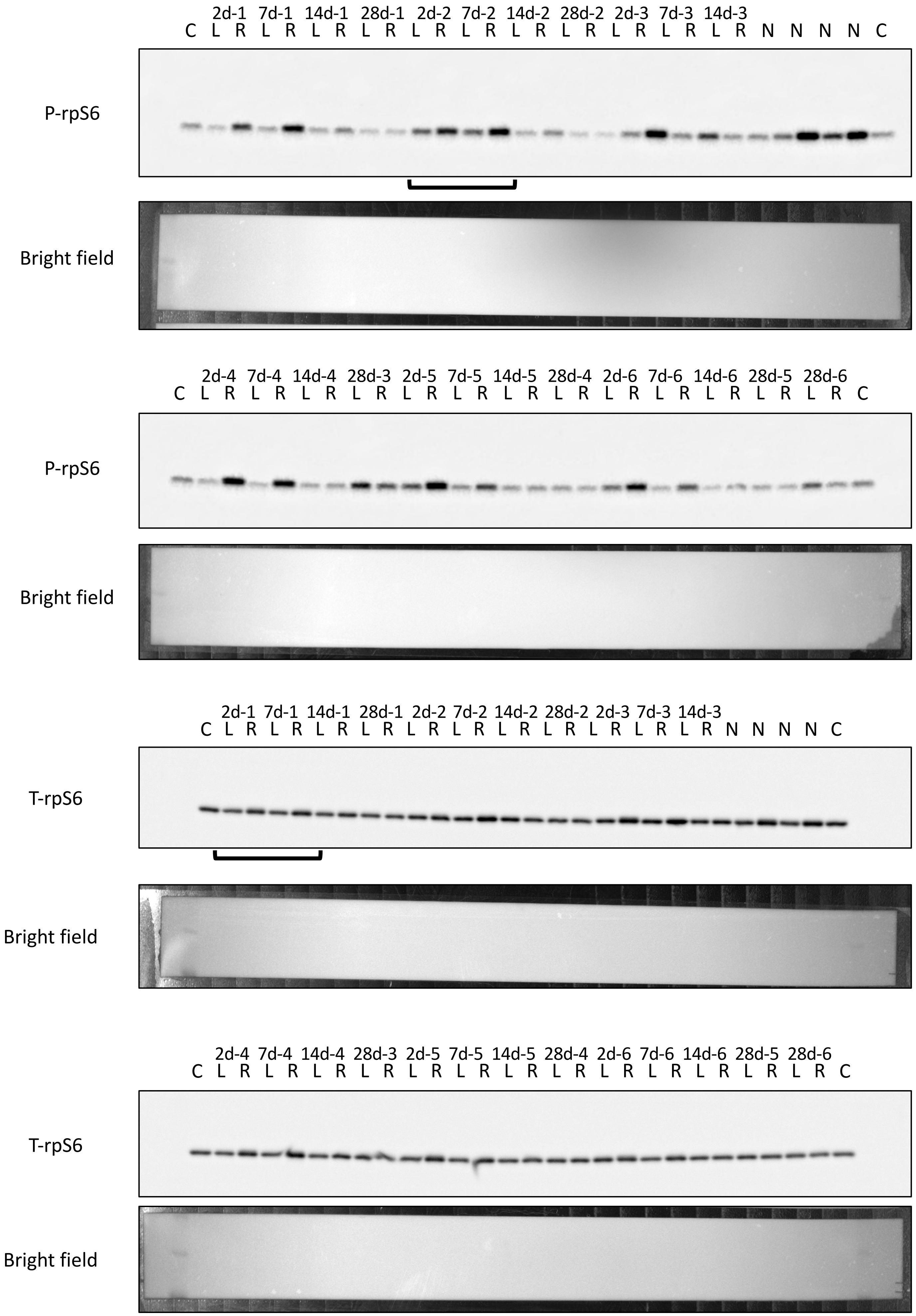
2d-1 7d-1 14d-1 28d-1 2d-2 7d-2 14d-2 28d-2 2d-3 7d-3 14d-3
C L R L R L R L R L R L R L R L R L R L R L R N N N N C
P-rpS6
Bright field
2d-4 7d-4 14d-4 28d-3 2d-5 7d-5 14d-5 28d-4 2d-6 7d-6 14d-6 28d-5 28d-6
C L R L R L R L R L R L R L R L R L R L R L R L R L R C
P-rpS6
Bright field
2d-1 7d-1 14d-1 28d-1 2d-2 7d-2 14d-2 28d-2 2d-3 7d-3 14d-3
C L R L R L R L R L R L R L R L R L R L R L R N N N N C
T-rpS6
Bright field
2d-4 7d-4 14d-4 28d-3 2d-5 7d-5 14d-5 28d-4 2d-6 7d-6 14d-6 28d-5 28d-6
C L R L R L R L R L R L R L R L R L R L R L R L R L R C
T-rpS6
Bright field

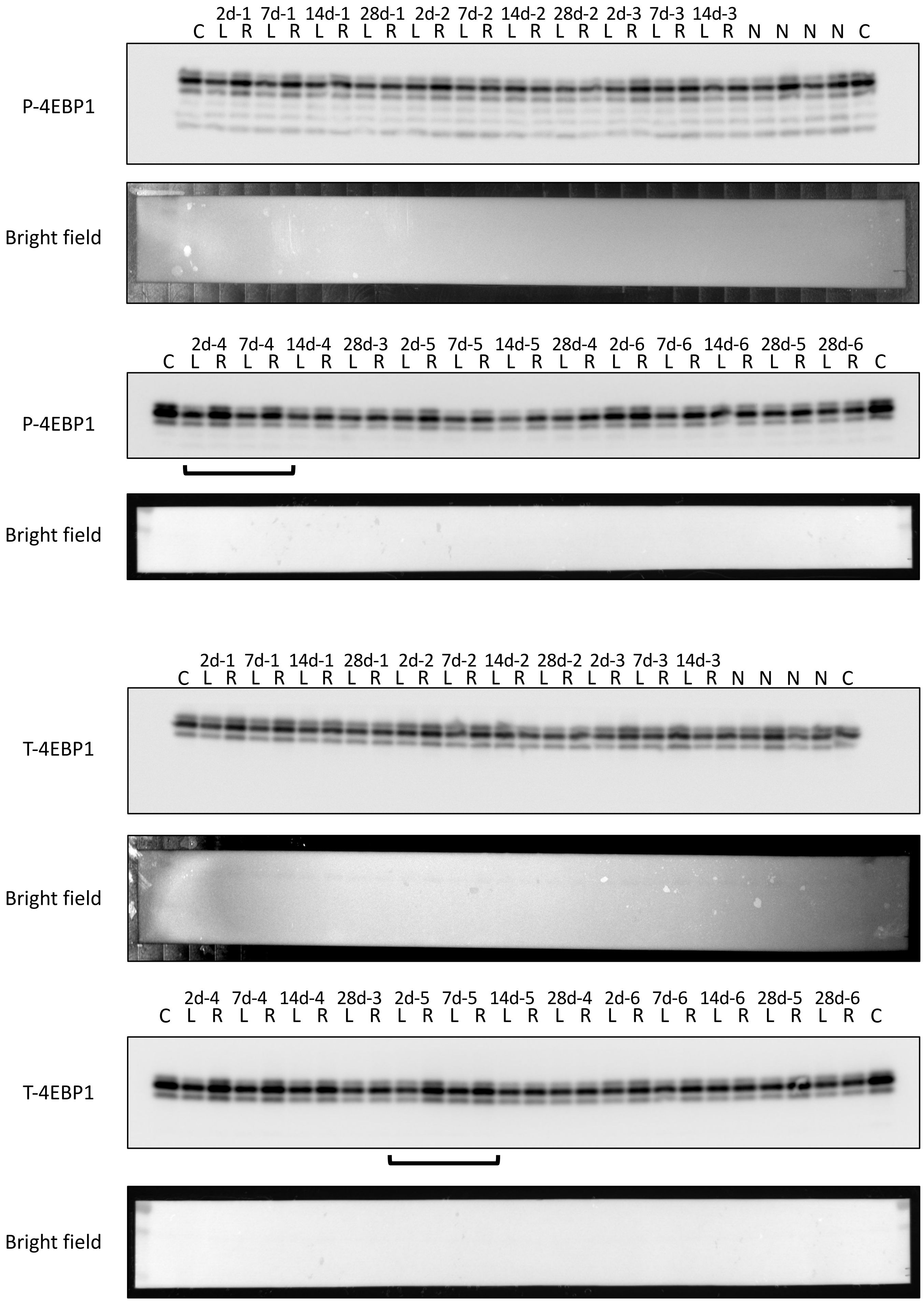
2d-1 7d-1 14d-1 28d-1 2d-2 7d-2 14d-2 28d-2 2d-3 7d-3 14d-3
C L R L R L R L R L R L R L R L R L R L R L R N N N N C
P-4EBP1
Bright field
2d-4 7d-4 14d-4 28d-3 2d-5 7d-5 14d-5 28d-4 2d-6 7d-6 14d-6 28d-5 28d-6
C L R L R L R L R L R L R L R L R L R L R L R L R L R C
P-4EBP1
Bright field
2d-1 7d-1 14d-1 28d-1 2d-2 7d-2 14d-2 28d-2 2d-3 7d-3 14d-3
C L R L R L R L R L R L R L R L R L R L R L R N N N N C
T-4EBP1
Bright field
2d-4 7d-4 14d-4 28d-3 2d-5 7d-5 14d-5 28d-4 2d-6 7d-6 14d-6 28d-5 28d-6
C L R L R L R L R L R L R L R L R L R L R L R L R L R C
T-4EBP1
Bright field

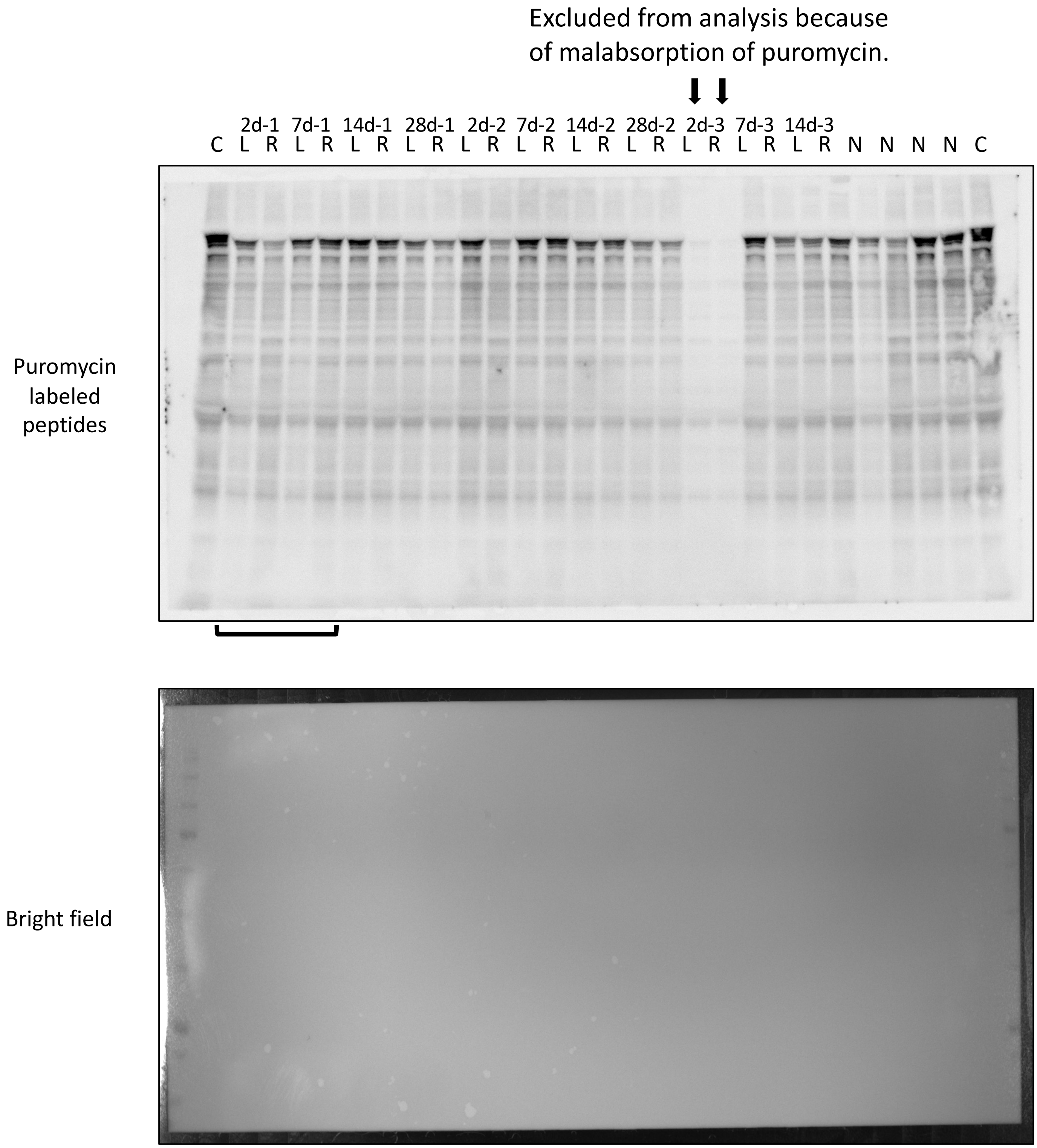
Excluded from analysis because of malabsorption of puromycin.
2d-1 7d-1 14d-1 28d-1 2d-2 7d-2 14d-2 28d-2 2d-3 7d-3 14d-3
C L R L R L R L R L R L R L R L R L R L R L R N N N N C
Puromycin labeled peptides
Bright field

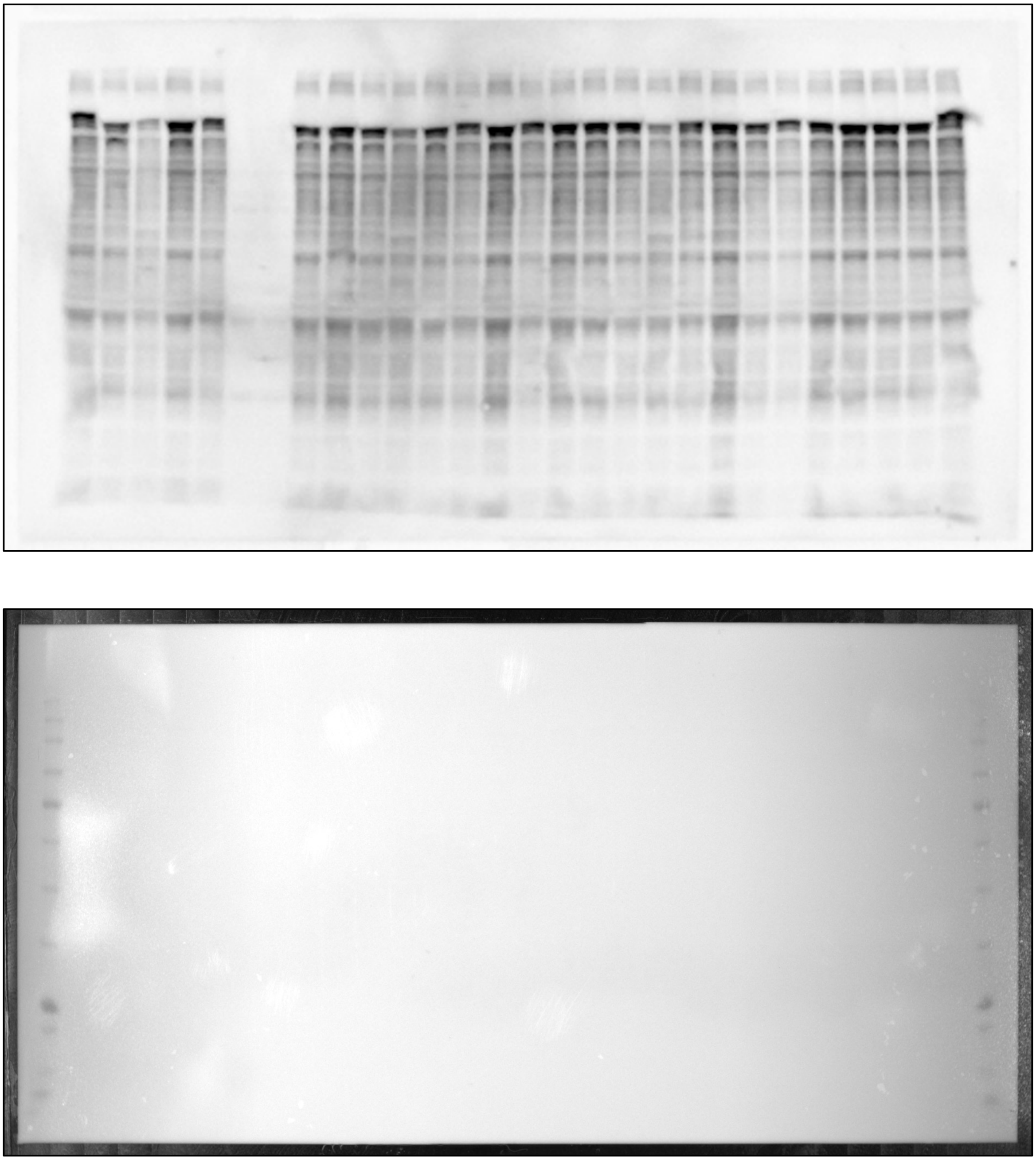
2d-4 7d-4 14d-4 28d-3 2d-5 7d-5 14d-5 28d-4 2d-6 7d-6 14d-6 28d-5 28d-6
C L R L R L R L R L R L R L R L R L R L R L R L R L R C
Puromycin labeled peptides
Bright field

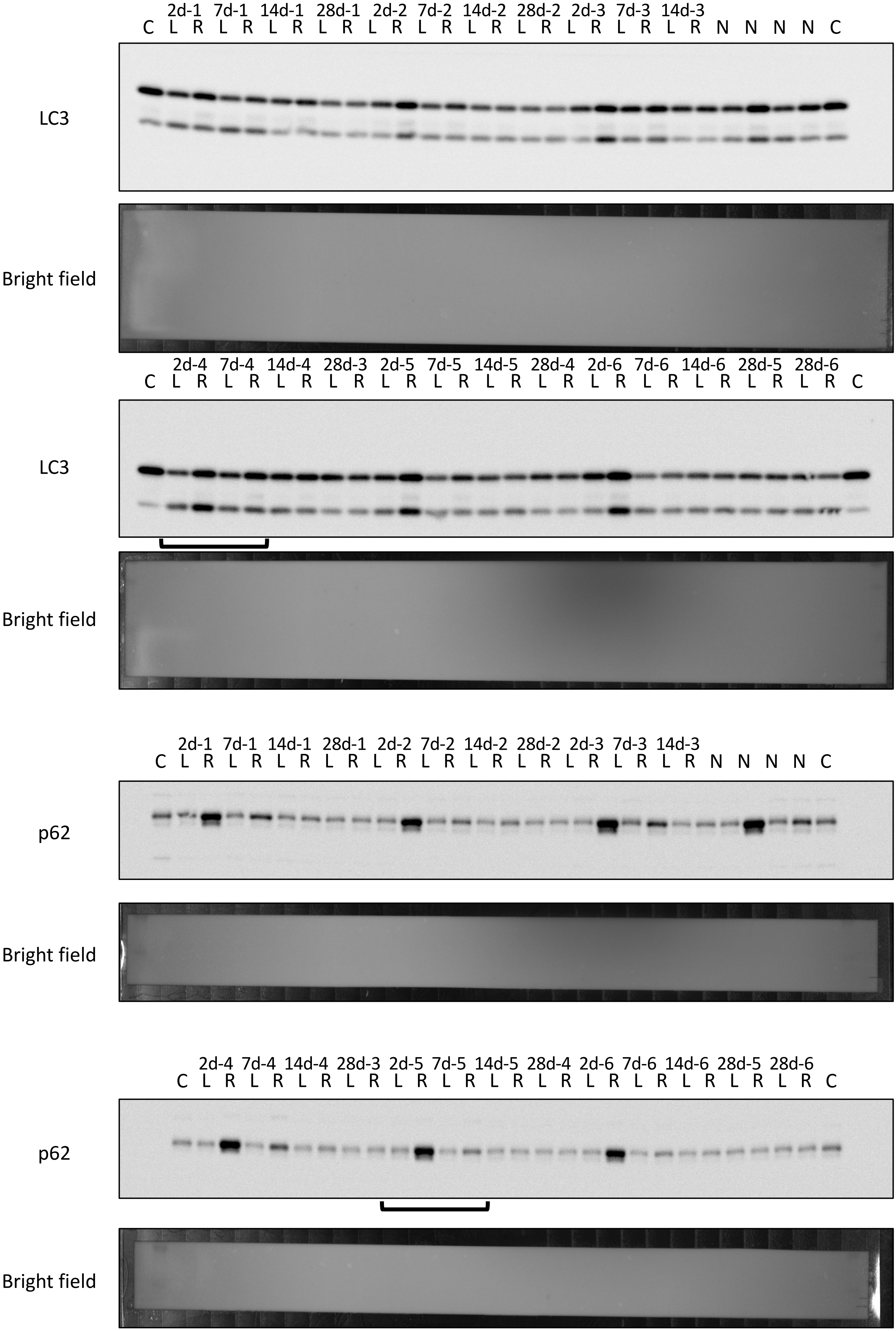

2d-1 7d-1 14d-1 28d-1 2d-2 7d-2 14d-2 28d-2 2d-3 7d-3 14d-3
C L R L R L R L R L R L R L R L R L R L R L R N N N N C
LC3
Bright field
2d-4 7d-4 14d-4 28d-3 2d-5 7d-5 14d-5 28d-4 2d-6 7d-6 14d-6 28d-5 28d-6
C L R L R L R L R L R L R L R L R L R L R L R L R L R C
LC3
Bright field
2d-1 7d-1 14d-1 28d-1 2d-2 7d-2 14d-2 28d-2 2d-3 7d-3 14d-3
C L R L R L R L R L R L R L R L R L R L R L R N N N N C
p62
Bright field
2d-4 7d-4 14d-4 28d-3 2d-5 7d-5 14d-5 28d-4 2d-6 7d-6 14d-6 28d-5 28d-6
C L R L R L R L R L R L R L R L R L R L R L R L R L R C
p62
Bright field

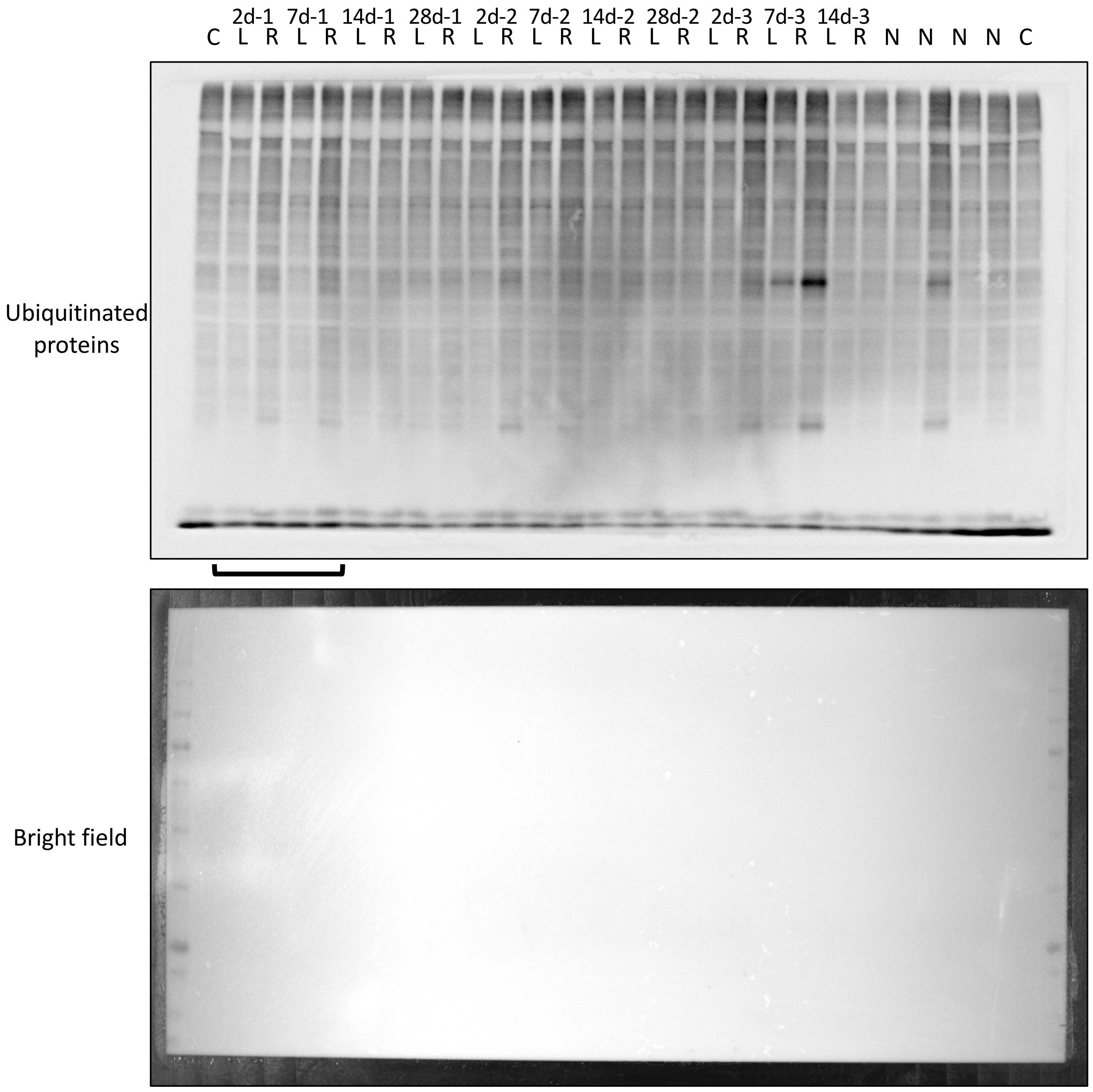
2d-1 7d-1 14d-1 28d-1 2d-2 7d-2 14d-2 28d-2 2d-3 7d-3 14d-3
C L R L R L R L R L R L R L R L R L R L R L R N N N N C
Ubiquitinated proteins
Bright field

Ubiquitinated proteins

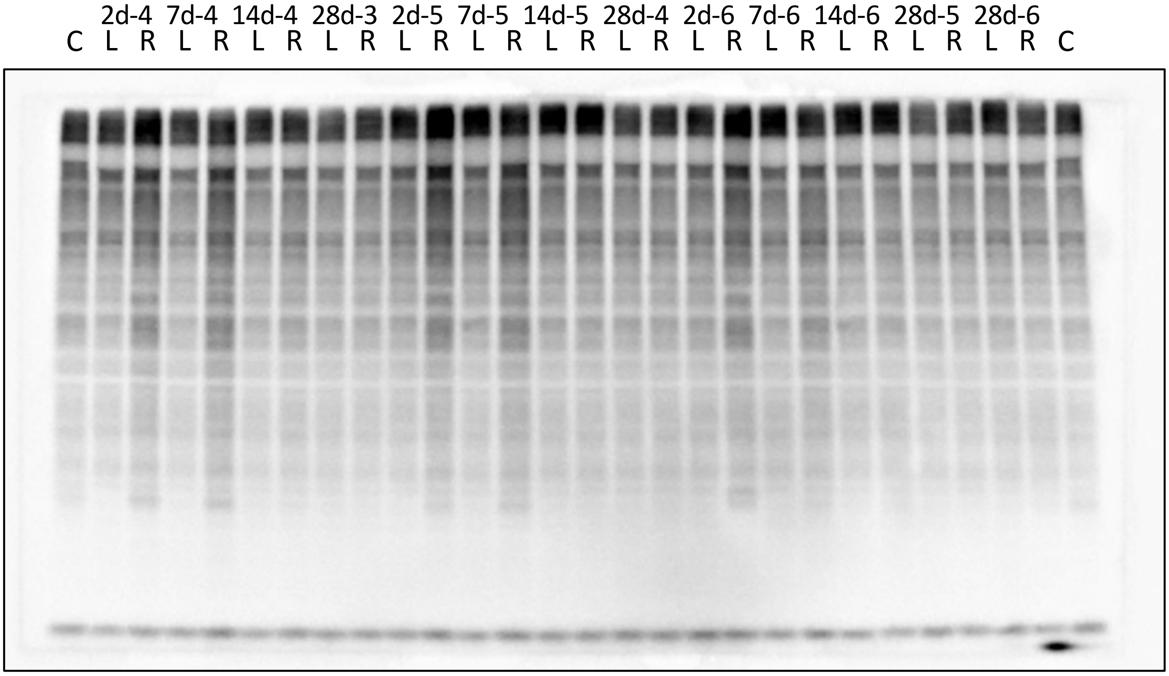

Bright field

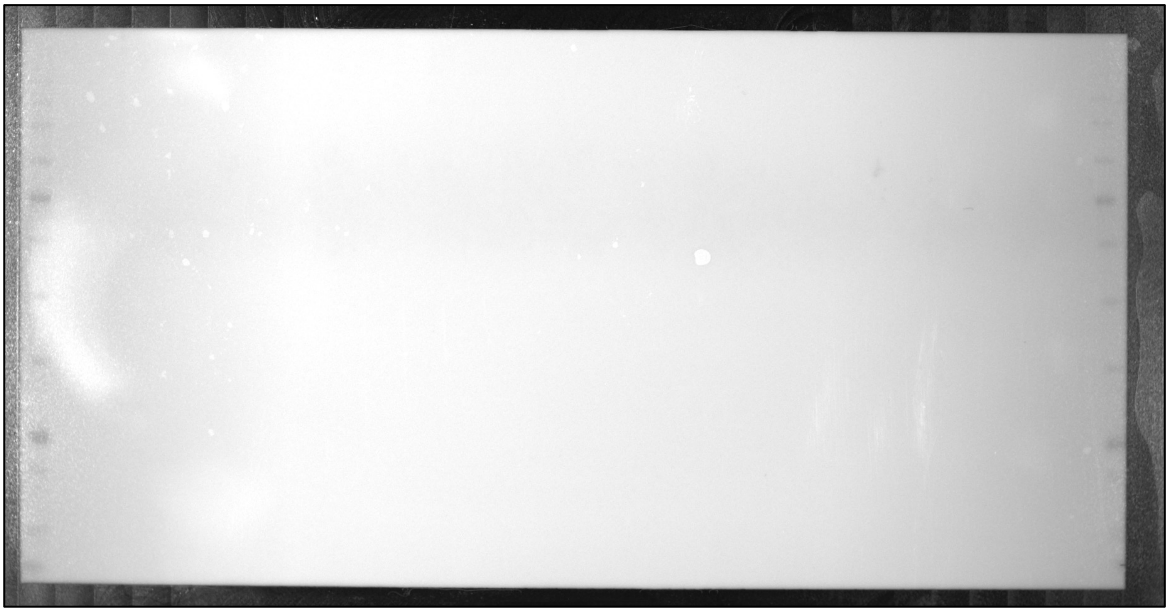

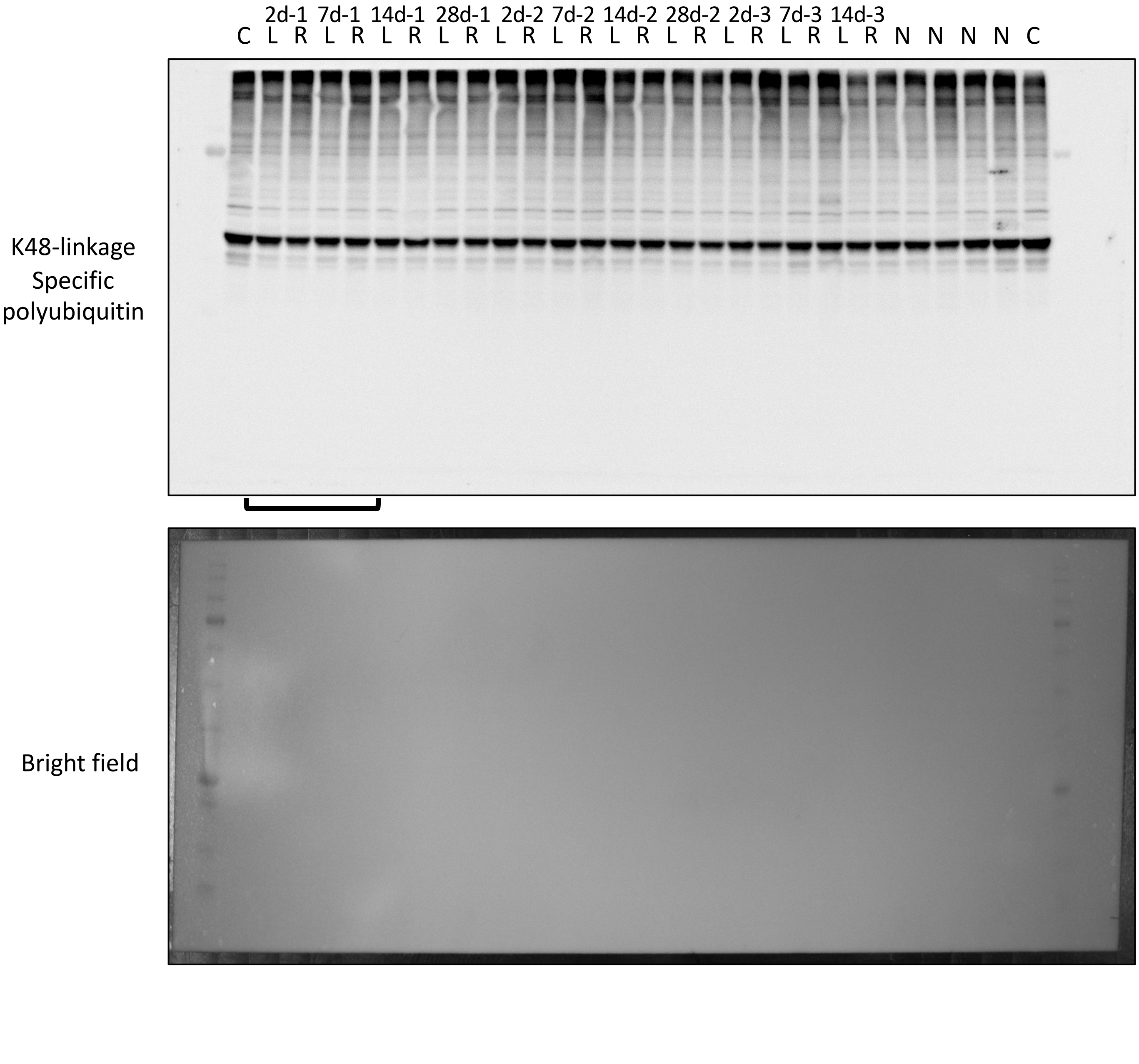
2d-1 7d-1 14d-1 28d-1 2d-2 7d-2 14d-2 28d-2 2d-3 7d-3 14d-3
C L R L R L R L R L R L R L R L R L R L R L R N N N N C
K48-linkage Specific polyubiquitin
Bright field

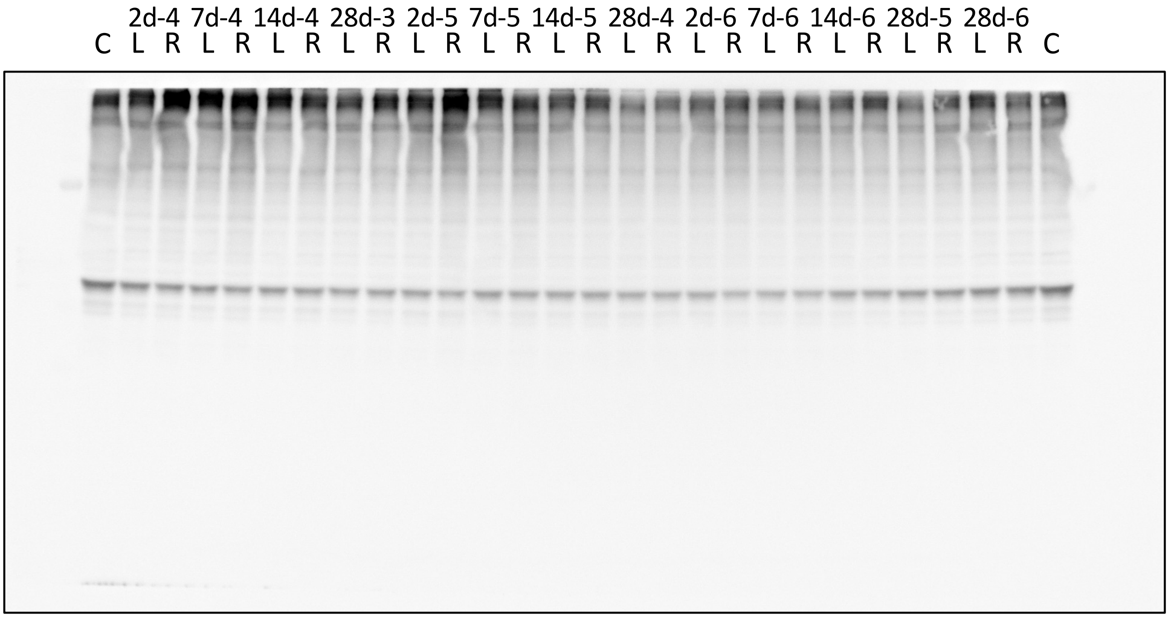

K48-linkage Specific polyubiquitin

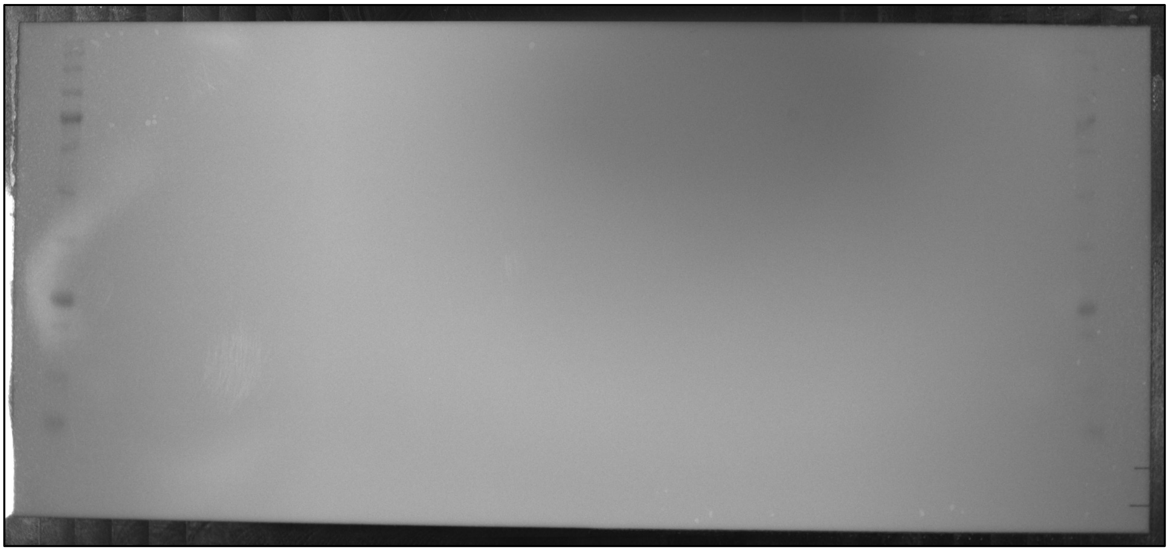

Bright field

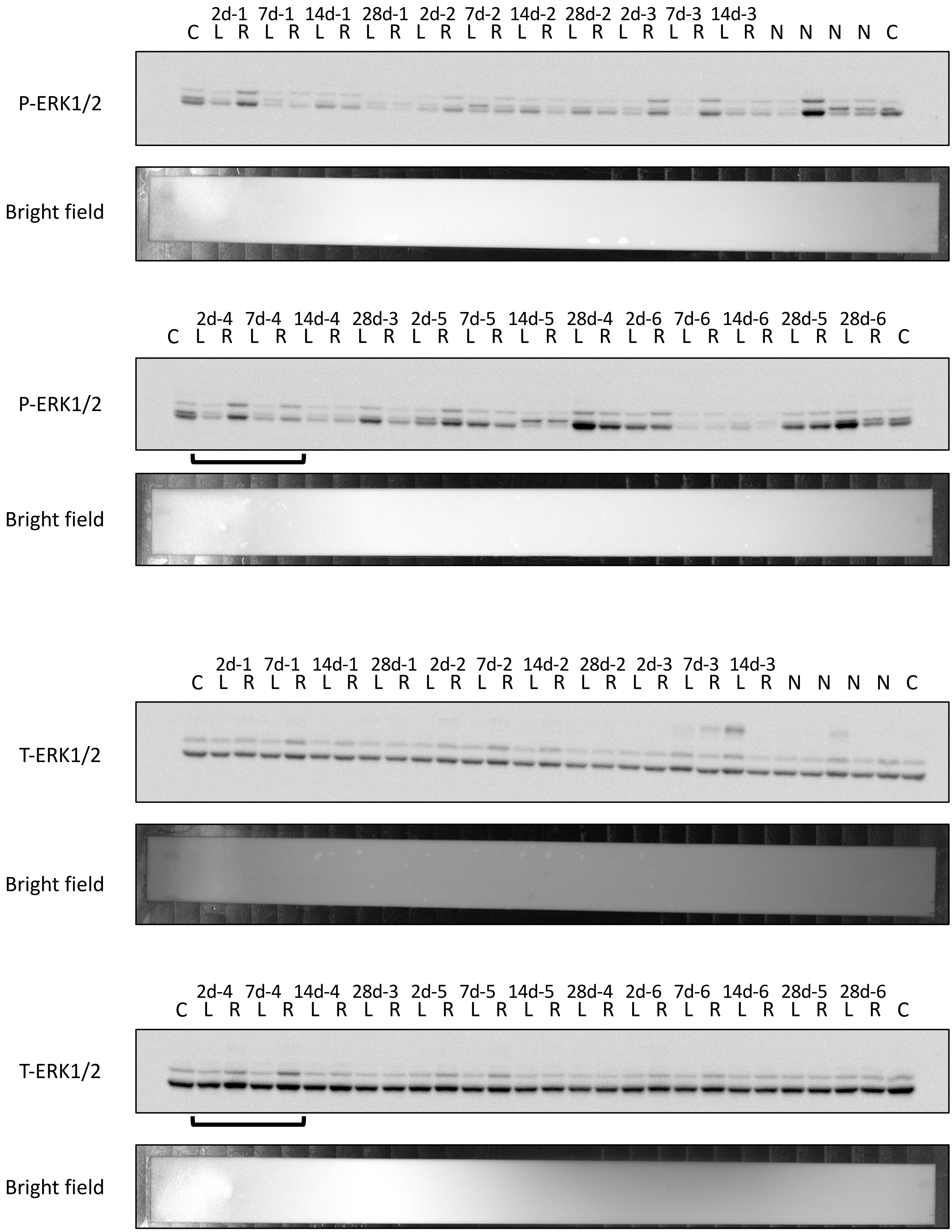
2d-1 7d-1 14d-1 28d-1 2d-2 7d-2 14d-2 28d-2 2d-3 7d-3 14d-3
C L R L R L R L R L R L R L R L R L R L R L R N N N N C
P-ERK1/2
Bright field
2d-4 7d-4 14d-4 28d-3 2d-5 7d-5 14d-5 28d-4 2d-6 7d-6 14d-6 28d-5 28d-6
C L R L R L R L R L R L R L R L R L R L R L R L R L R C
P-ERK1/2
Bright field
2d-1 7d-1 14d-1 28d-1 2d-2 7d-2 14d-2 28d-2 2d-3 7d-3 14d-3
C L R L R L R L R L R L R L R L R L R L R L R N N N N C
T-ERK1/2
Bright field
2d-4 7d-4 14d-4 28d-3 2d-5 7d-5 14d-5 28d-4 2d-6 7d-6 14d-6 28d-5 28d-6
C L R L R L R L R L R L R L R L R L R L R L R L R L R C
T-ERK1/2
Bright field

Ponceau S

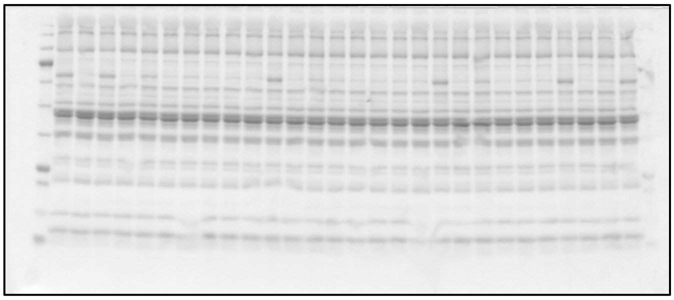

GFP_1

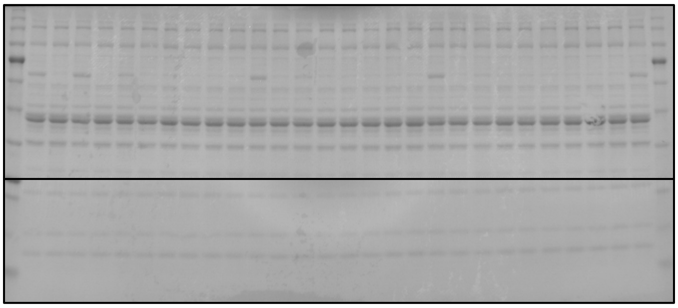

GFP_2, T-p70S6K_2, P-4EBP1_2*

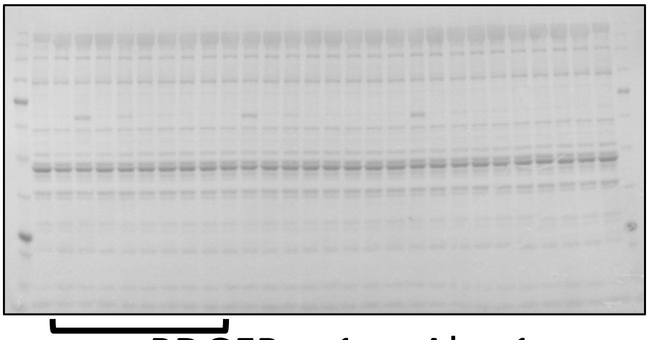

PDGFRα_1, p-Akt_1

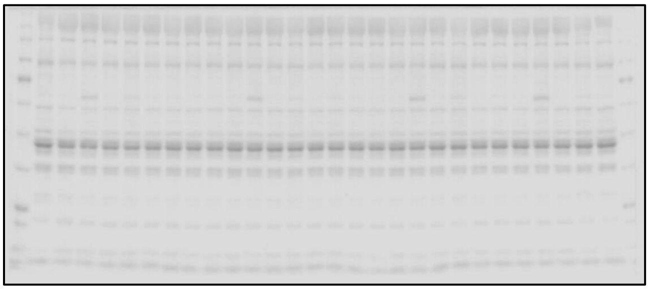

PDGFRα_2, P-Akt_2

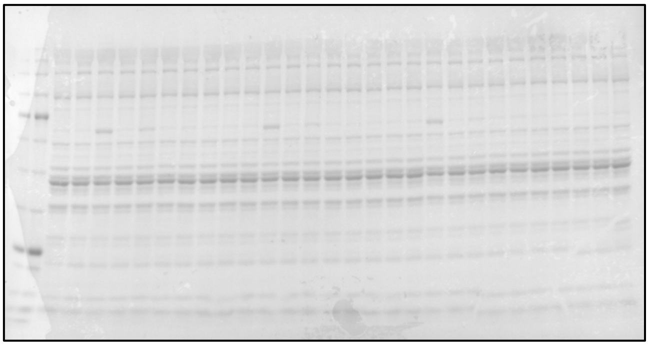

T-Akt_1

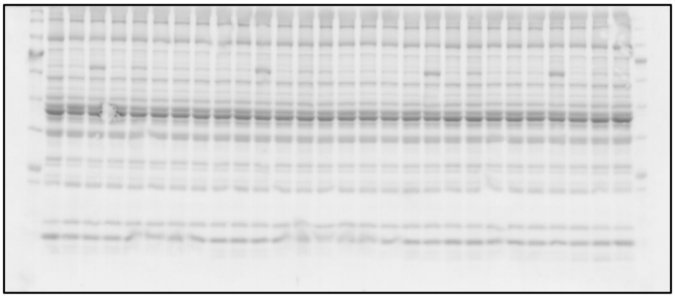

T-Akt_2

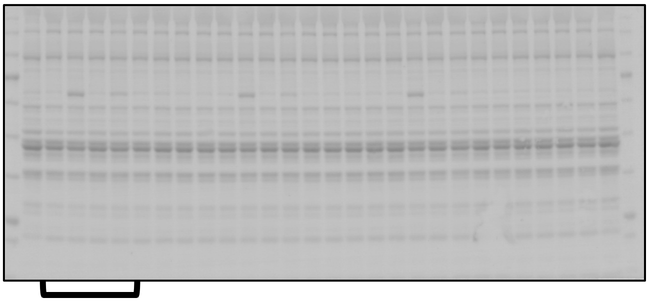

P-p70S6K_1, P-rpS6_1

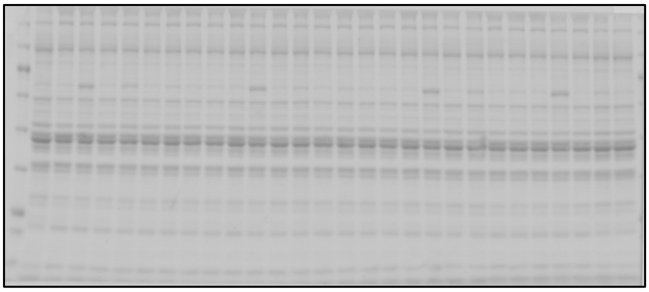

P-p70S6K_2, P-rpS6_2

Ponceau S

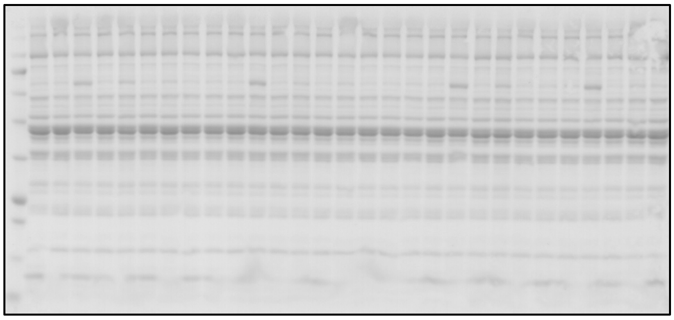

T-p70S6K_1

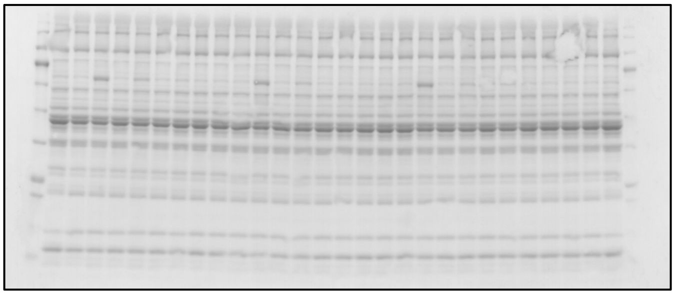

T-rpS6_1, T-4EBP1_1

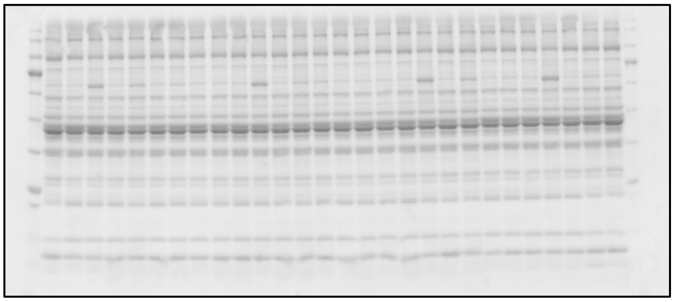

T-rpS6_2

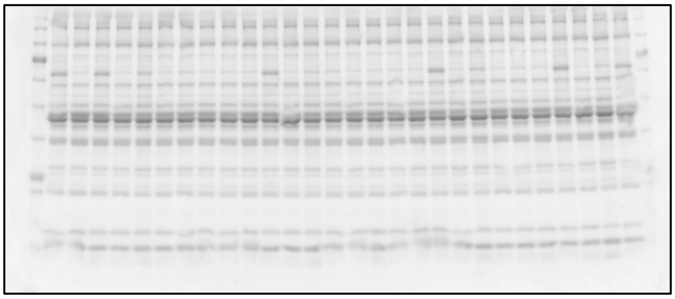

P-4EBP1_1

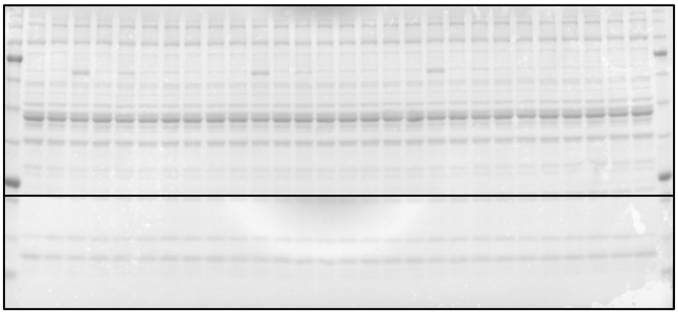

T-4EBP1_2*

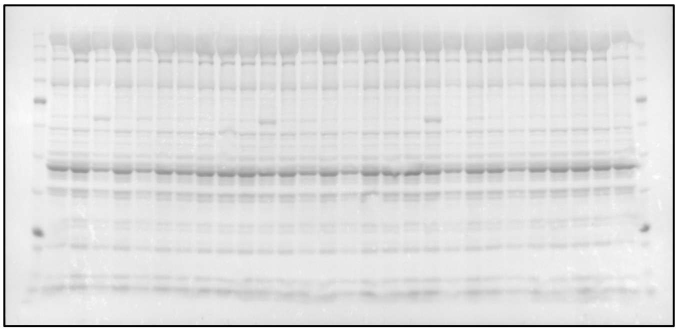

Puromycin_1

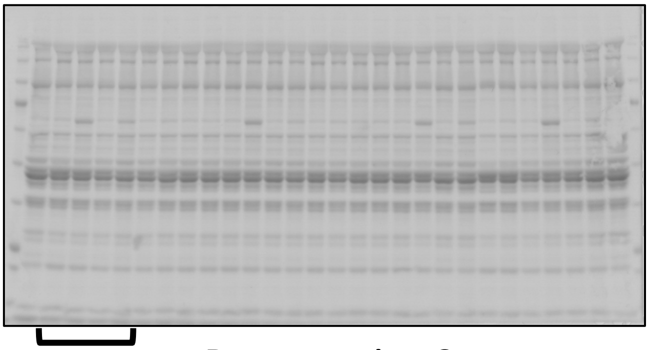

Puromycin_2

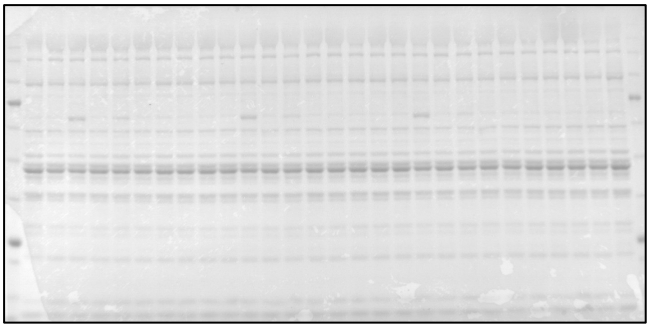

Ubiquitin_1

Ponceau S

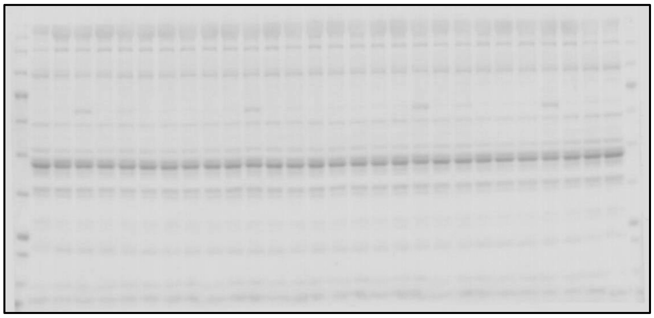

Ubiquitin_2

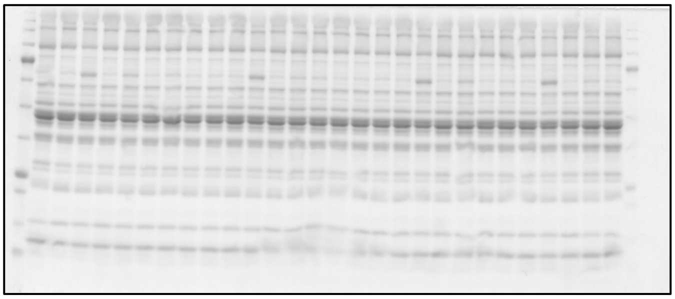

K48-linkage specific polyubiquitin_1

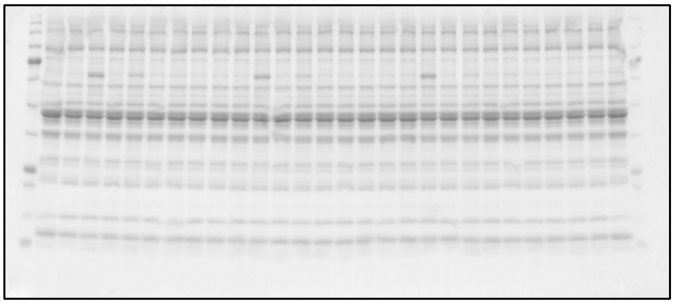

K48-linkage specific polyubiquitin_2

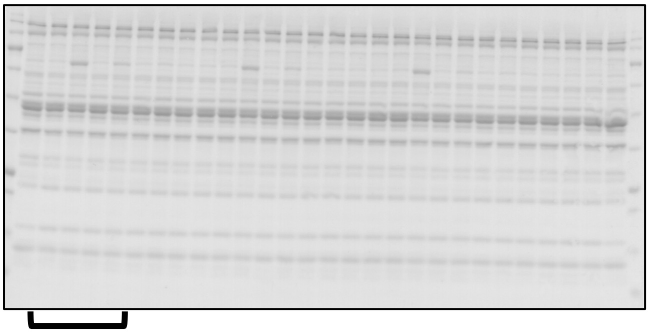

P-ERK_1, LC3_1

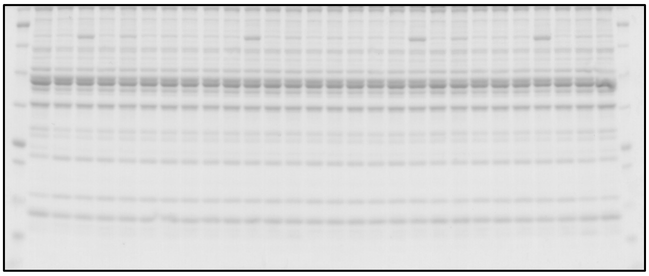

LC3_2

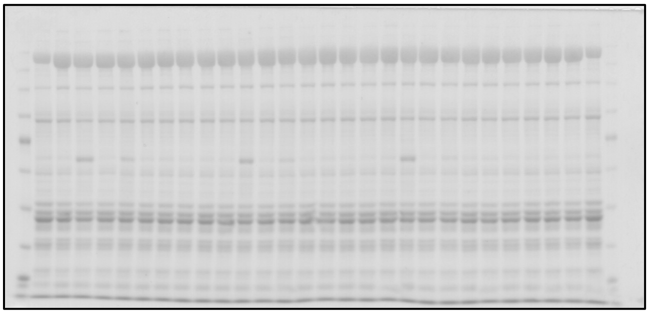

p62_1

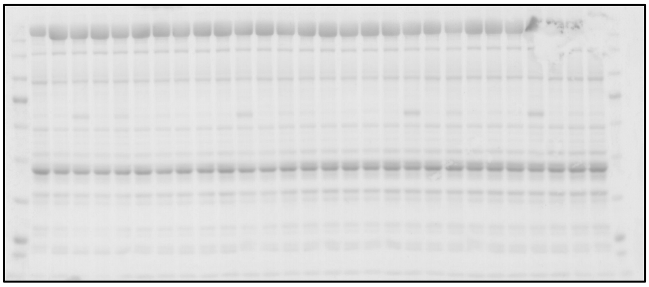

p62_2

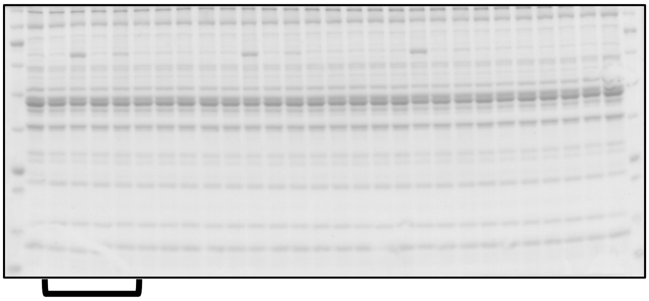

P-ERK_1

Ponceau S

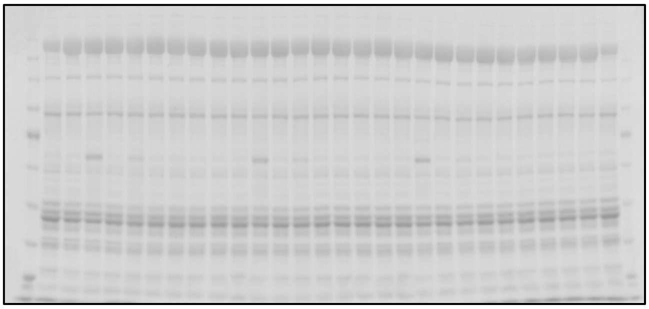

T-ERK_1

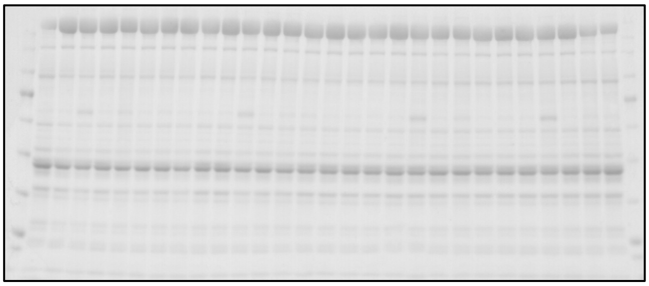

T-ERK_2

*Because of the camera did not work properly, we got 2 separate pictures.

Supplemental Information 2

Uncropped images used for histochemical analysis. Each images for same staining were taken under same condition. Injected: the area were likely injected with MSCs, distant: the area far from the point injected with MSCs.

Phase contrast and GFP (without staining)

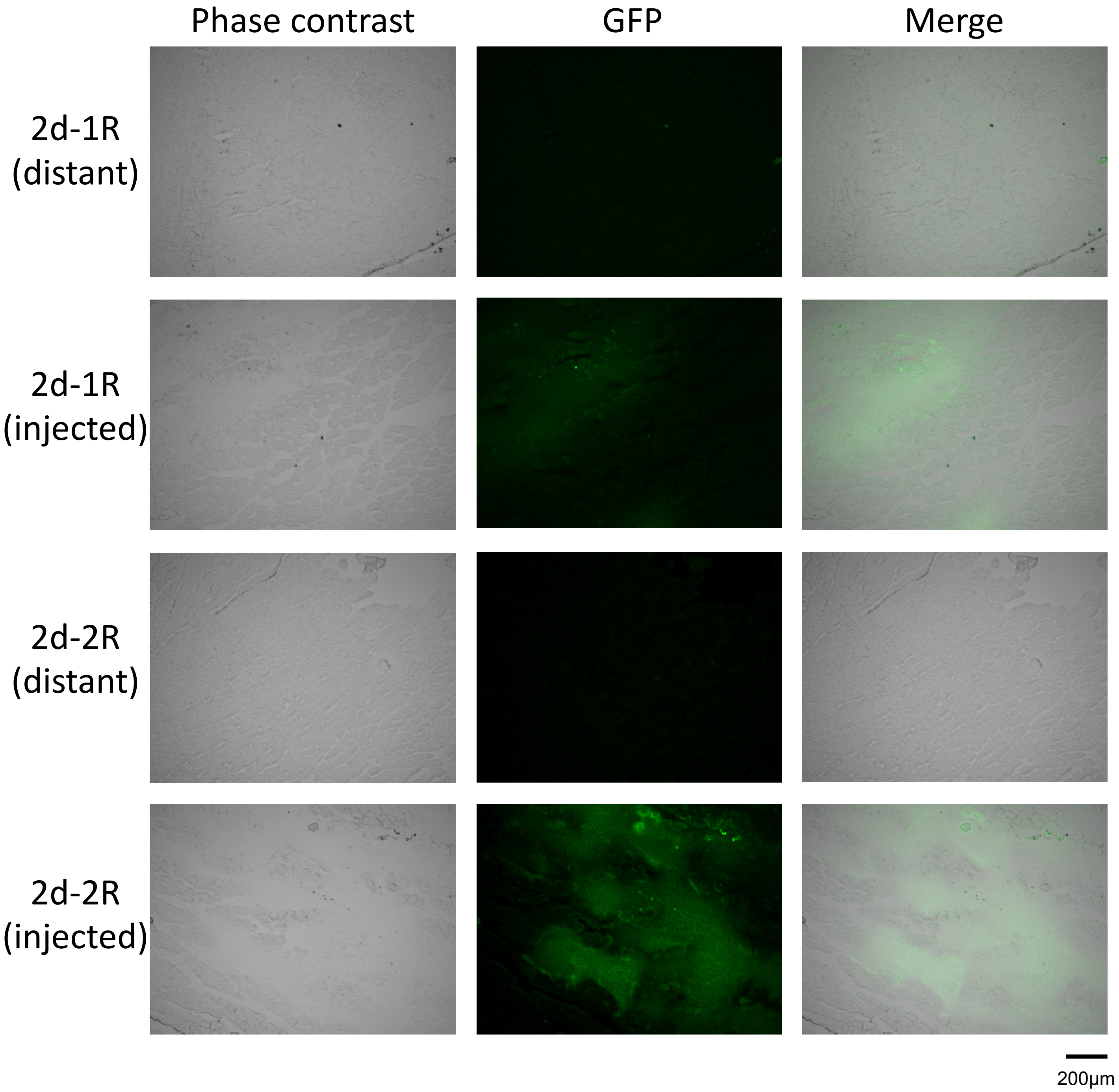

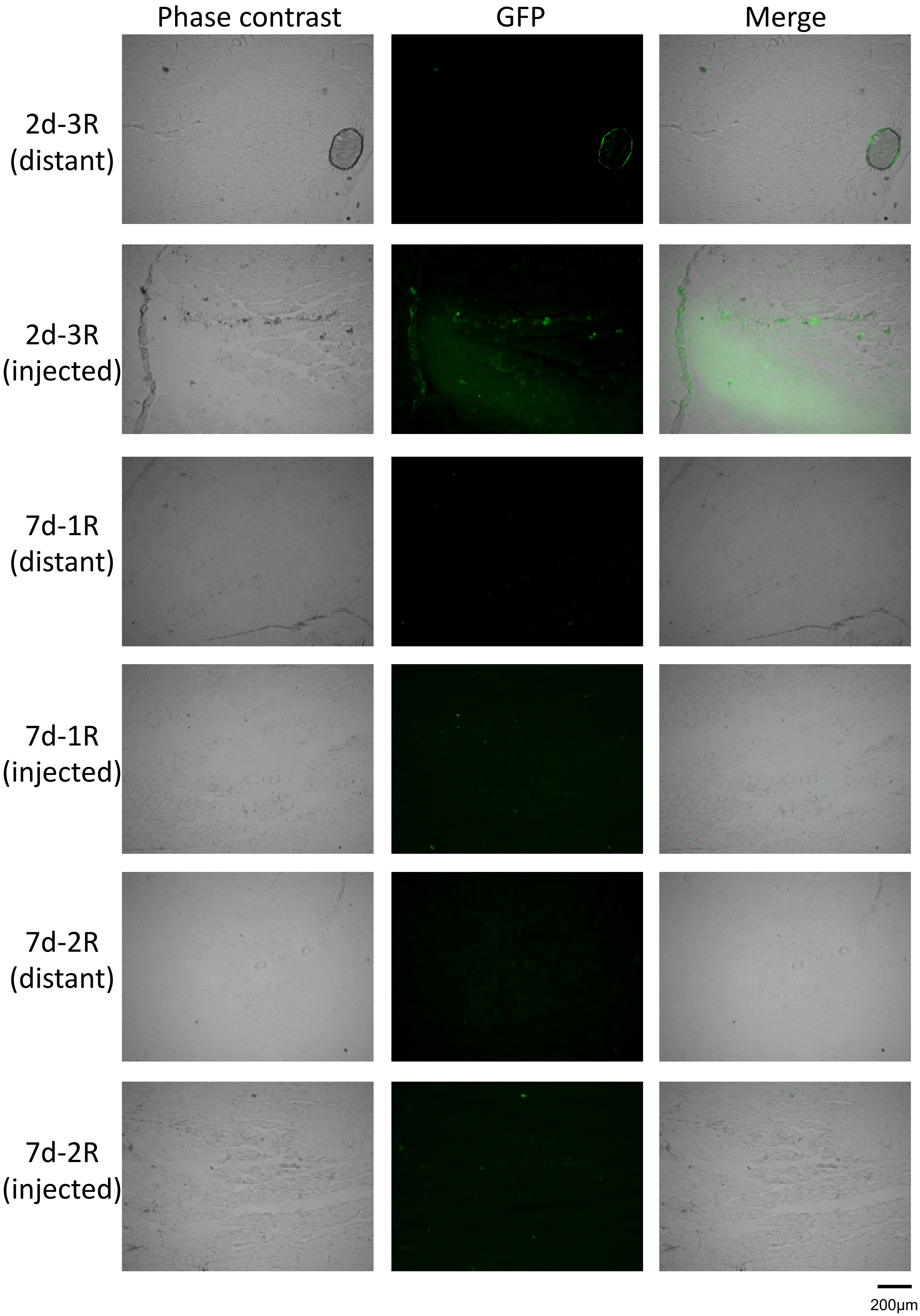
Phase contrast
GFP
Merge
2d-3R (distant)
2d-3R (injected)
7d-1R (distant)
7d-1R (injected)
7d-2R (distant)
7d-2R (injected)
200μm

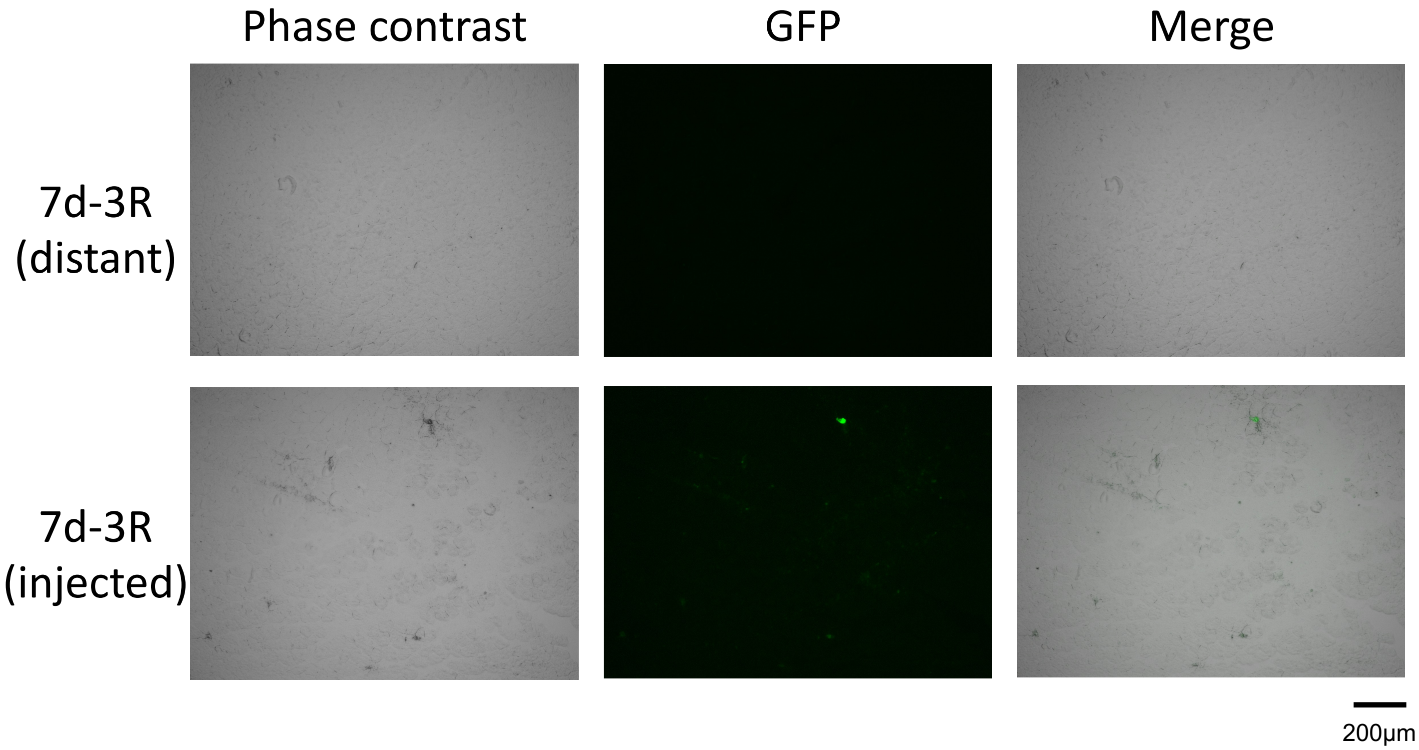

Phase contrast
GFP
Merge
7d-3R
(distant)
7d-3R
(injected)
200μm

Hematoxylin and Eosin staining

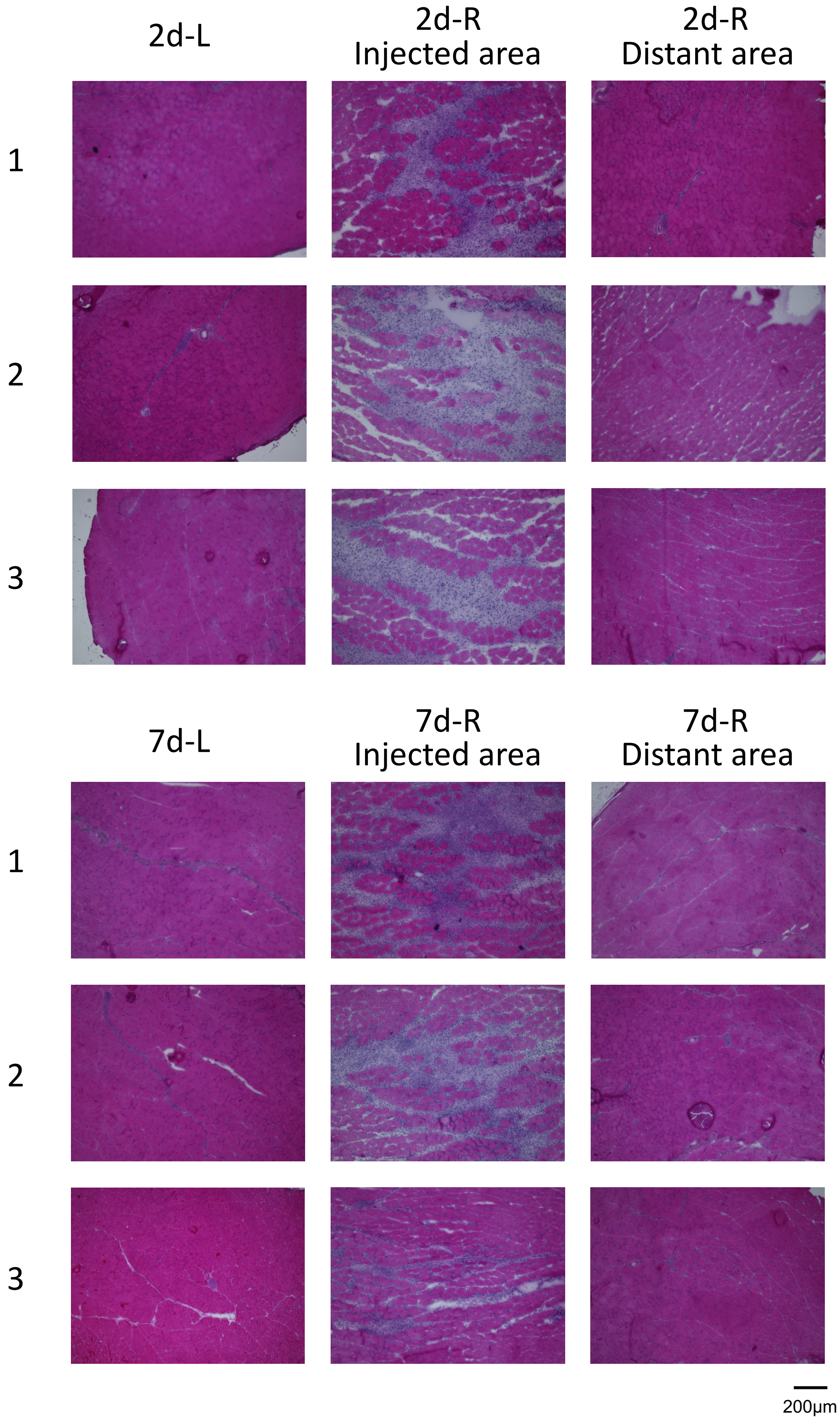

Whole section image of injected muscle (HE)

2d-1R

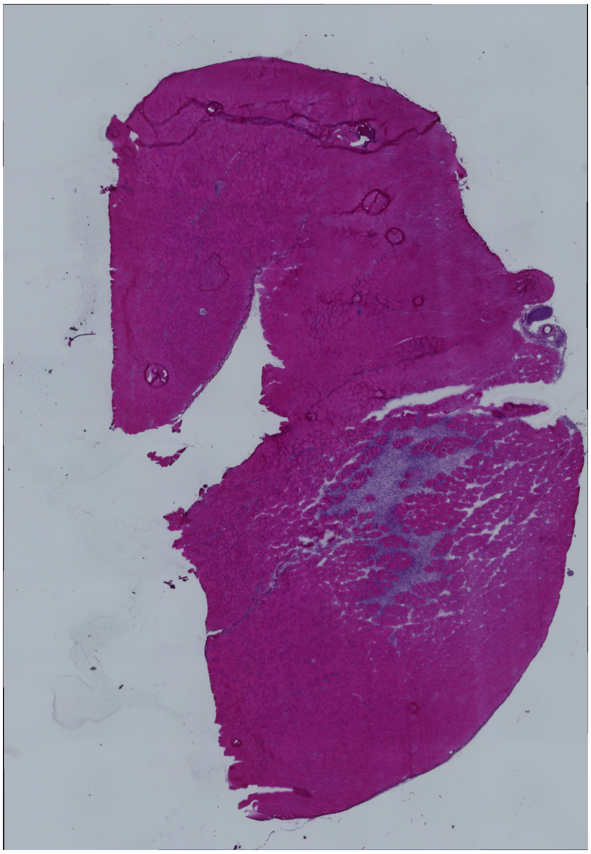

2d-2R

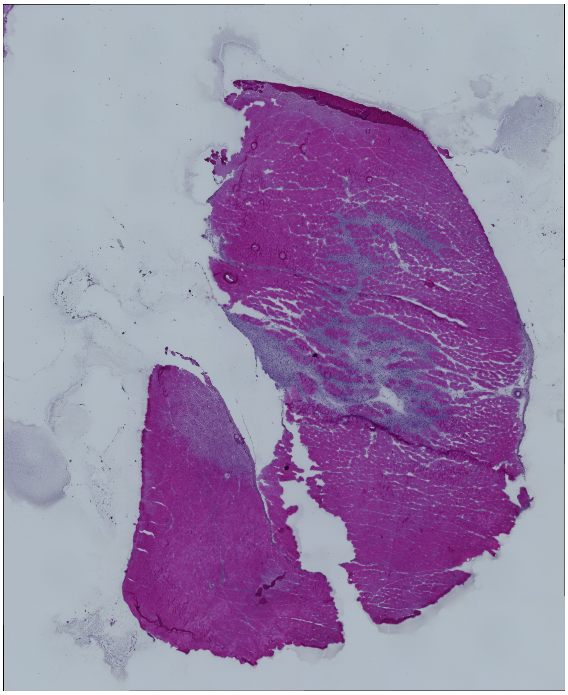

2d-3R

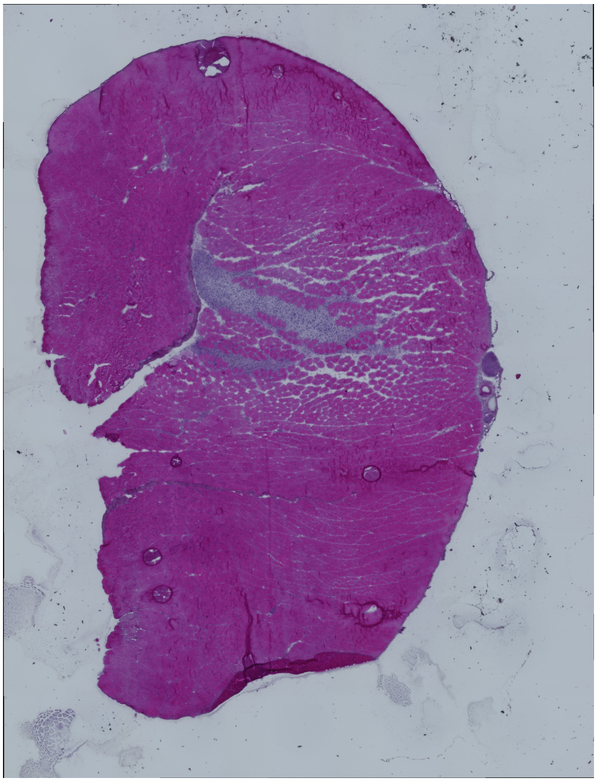

7d-1R

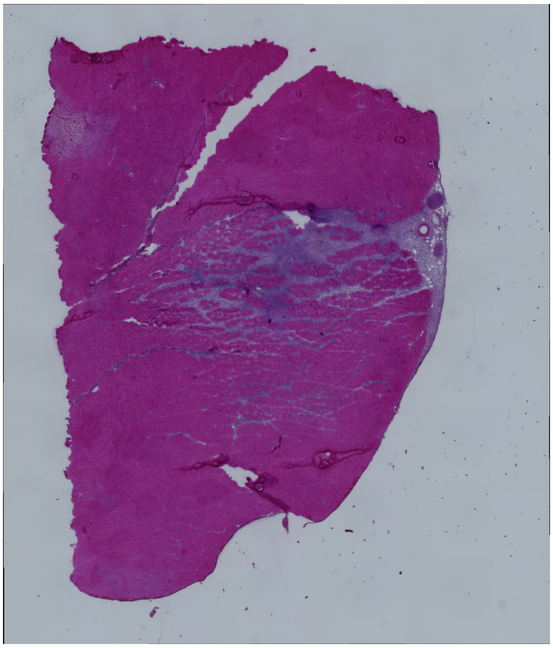

7d-2R

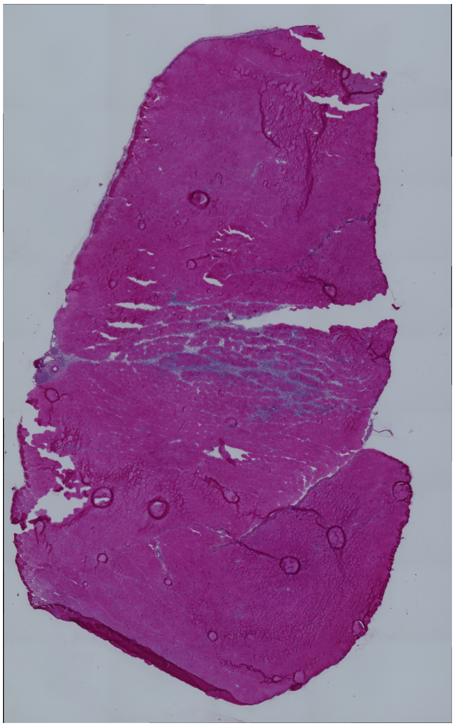

7d-3R

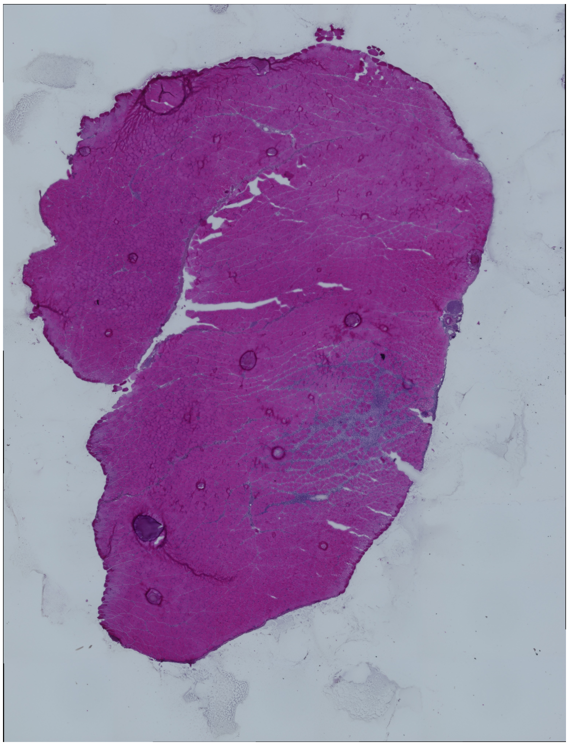

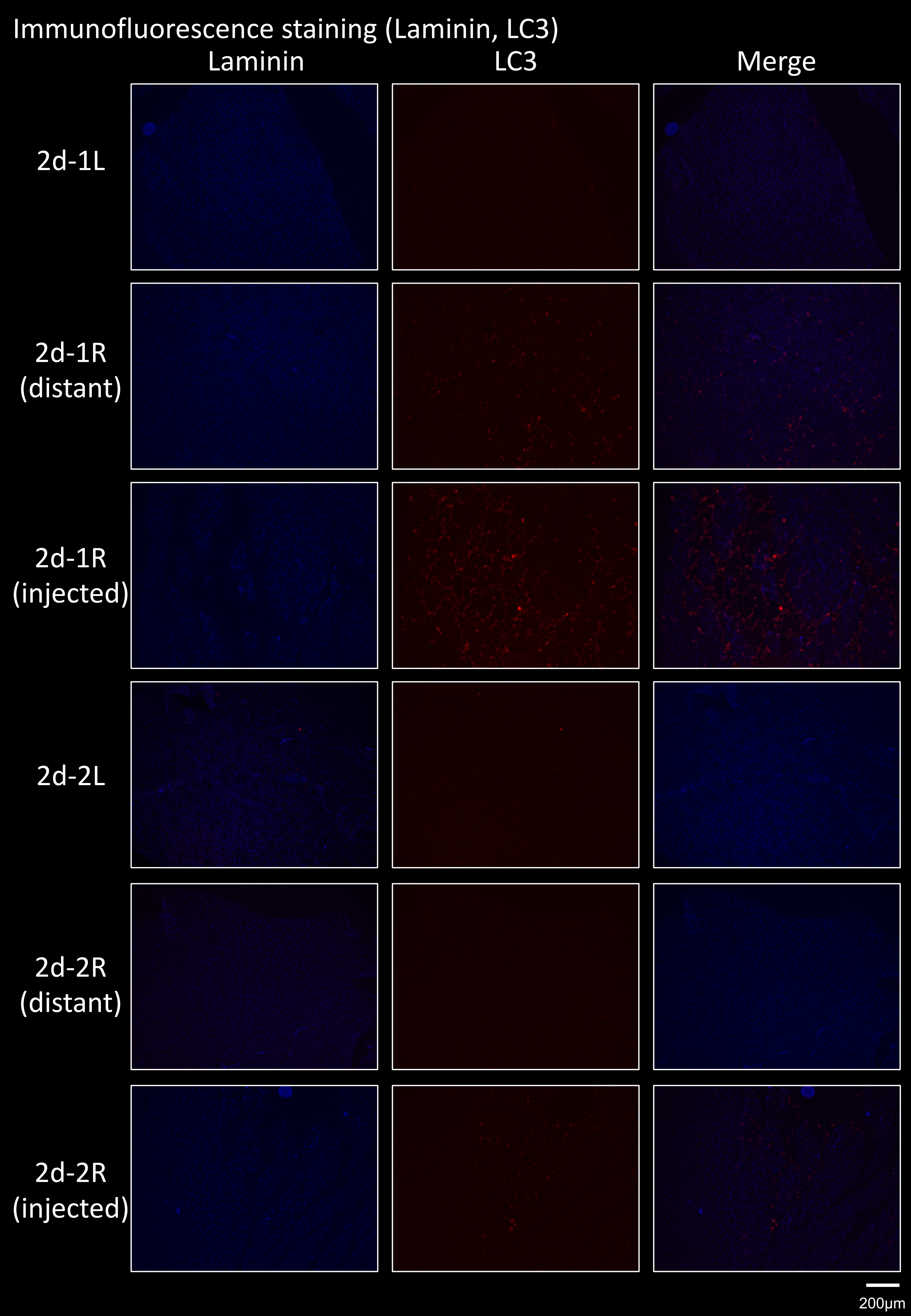
Immunofluorescence staining (Laminin, LC3)
Laminin
LC3
Merge
2d-1L
2d-1R (distant)
2d-1R (injected)
2d-2L
2d-2R (distant)
2d-2R (injected)
200μm

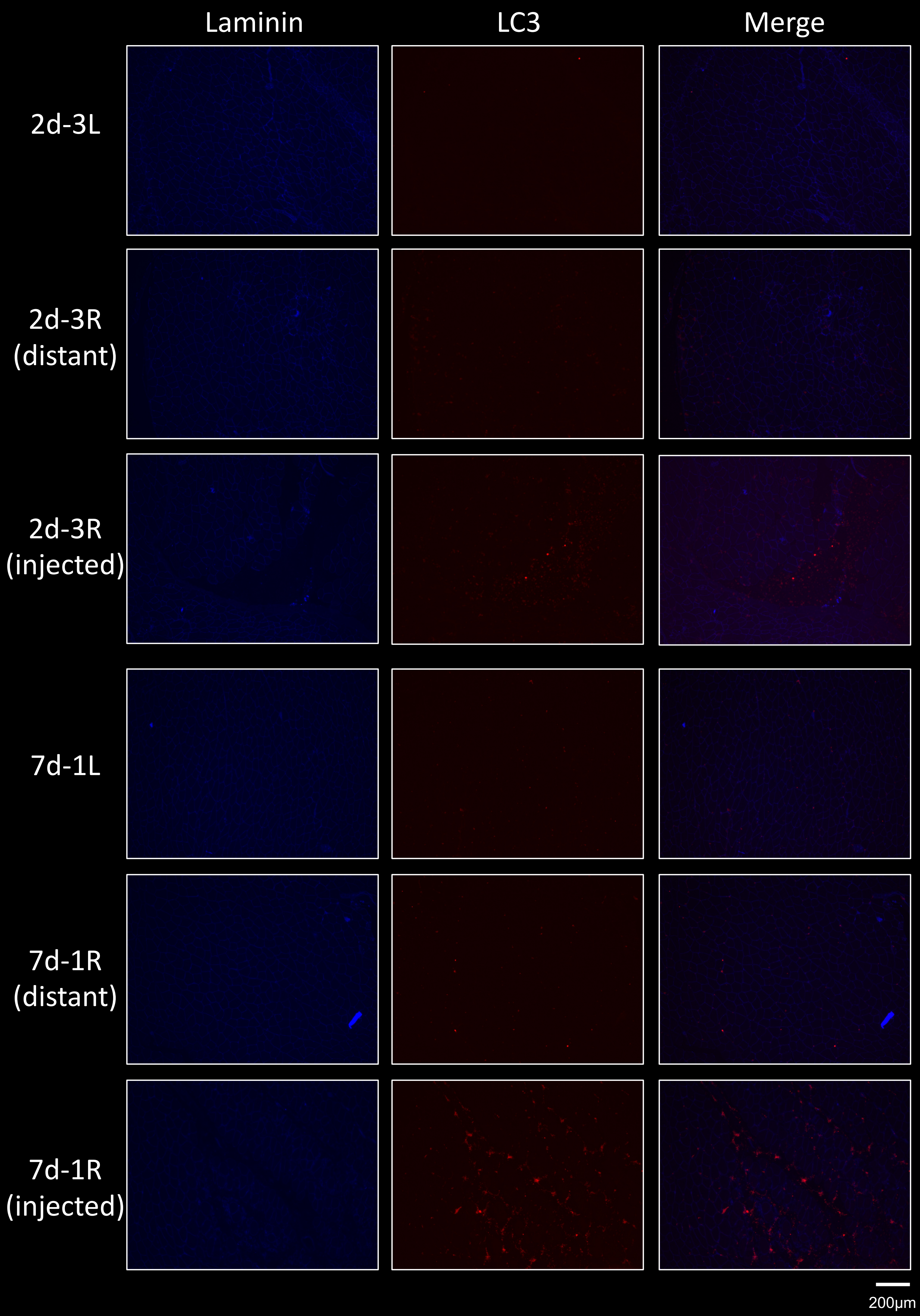
Laminin
LC3
Merge
2d-3L
2d-3R (distant)
2d-3R (injected)
7d-1L
7d-1R (distant)
7d-1R (injected)
200μm

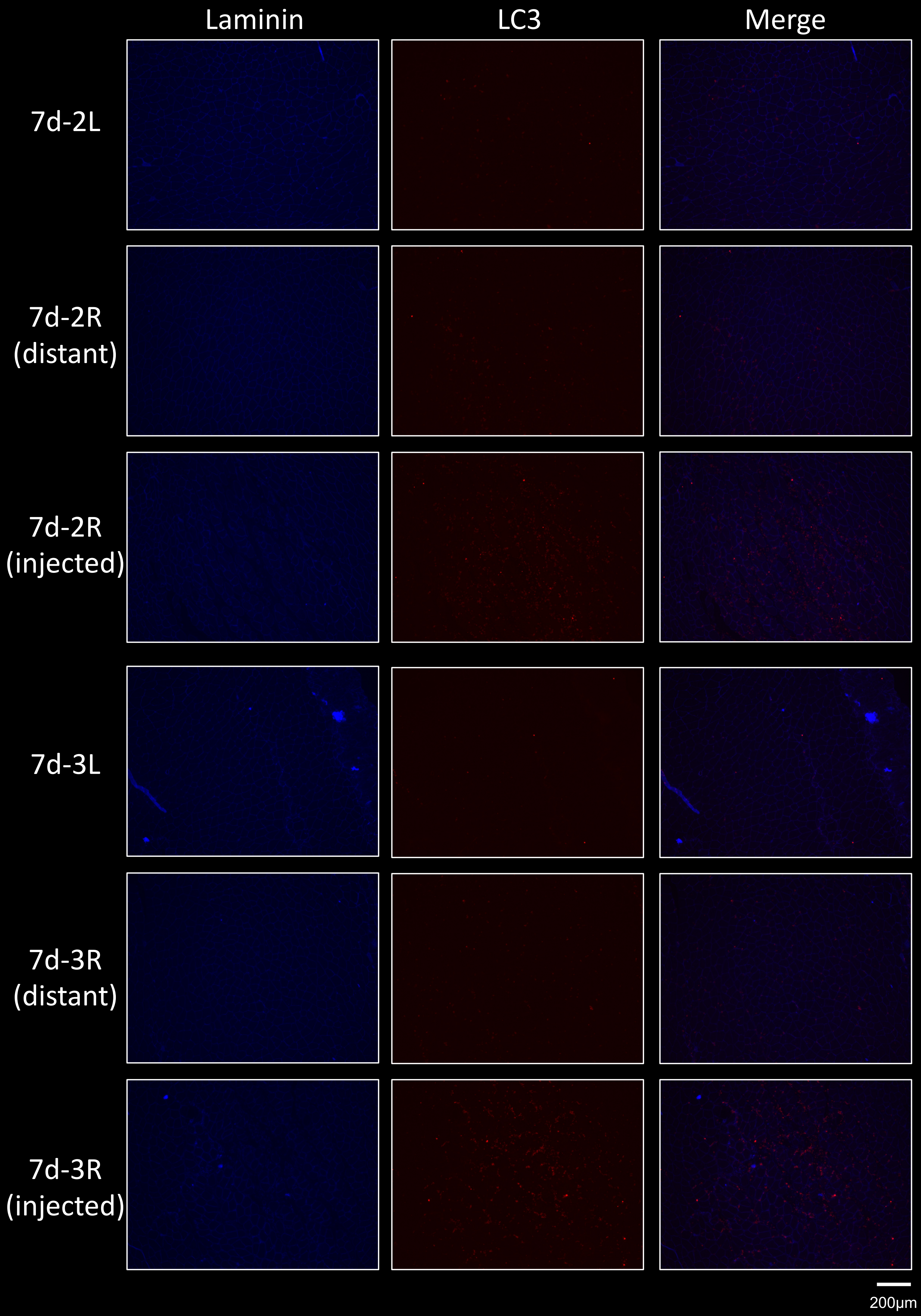
Laminin
LC3
Merge
7d-2L
7d-2R
(distant)
7d-2R
(injected)
7d-3L
7d-3R
(distant)
7d-3R
(injected)
200µm
